# Long-term participant retention and engagement patterns in an app and wearable-based multinational remote digital depression study

**DOI:** 10.1038/s41746-023-00749-3

**Published:** 2023-02-17

**Authors:** Yuezhou Zhang, Abhishek Pratap, Amos A. Folarin, Shaoxiong Sun, Nicholas Cummins, Faith Matcham, Srinivasan Vairavan, Judith Dineley, Yatharth Ranjan, Zulqarnain Rashid, Pauline Conde, Callum Stewart, Katie M. White, Carolin Oetzmann, Alina Ivan, Femke Lamers, Sara Siddi, Carla Hernández Rambla, Sara Simblett, Raluca Nica, David C. Mohr, Inez Myin-Germeys, Til Wykes, Josep Maria Haro, Brenda W. J. H. Penninx, Peter Annas, Vaibhav A. Narayan, Matthew Hotopf, Richard J. B. Dobson, RADAR-CNS consortium

**Affiliations:** 1 Institute of Psychiatry, Psychology and Neuroscience, King’s College London, London, UK; 2 Krembil Center for Neuroinformatics, CAMH, Toronto, ON Canada; 3 University of Toronto, Toronto, ON Canada; 4 University of Washington, Seattle, WA USA; 5 Davos Alzheimer’s Collaborative, Geneva, Switzerland; 6 University College London, London, UK; 7 South London and Maudsley NHS Foundation Trust, London, UK; 8 Health Data Research UK London, University College London, London, UK; 9 School of Psychology, University of Sussex, Falmer, East Sussex, UK; 10 Janssen Research and Development, LLC, Titusville, NJ USA; 11 Department of Psychiatry and Amsterdam Public Health Research Institute, Amsterdam UMC, Vrije Universiteit, Amsterdam, The Netherlands; 12 Parc Sanitari Sant Joan de Déu, Fundació Sant Joan de Déu, CIBERSAM, Universitat de Barcelona, Barcelona, Spain; 13 RADAR-CNS Patient Advisory Board, King’s College London, London, UK; 14 The Romanian League for Mental Health, Bucharest, Romania; 15 Center for Behavioral Intervention Technologies, Department of Preventative Medicine, Northwestern University, Chicago, IL USA; 16 Katholieke Universiteit Leuven, Leuven, Belgium; 17 H. Lundbeck A/S, Copenhagen, Denmark

**Keywords:** Depression, Bioinformatics

## Abstract

Recent growth in digital technologies has enabled the recruitment and monitoring of large and diverse populations in remote health studies. However, the generalizability of inference drawn from remotely collected health data could be severely impacted by uneven participant engagement and attrition over the course of the study. We report findings on long-term participant retention and engagement patterns in a large multinational observational digital study for depression containing active (surveys) and passive sensor data collected via Android smartphones, and Fitbit devices from 614 participants for up to 2 years. Majority of participants (67.6%) continued to remain engaged in the study after 43 weeks. Unsupervised clustering of participants’ study apps and Fitbit usage data showed 3 distinct engagement subgroups for each data stream. We found: (i) the least engaged group had the highest depression severity (4 PHQ8 points higher) across all data streams; (ii) the least engaged group (completed 4 bi-weekly surveys) took significantly longer to respond to survey notifications (3.8 h more) and were 5 years younger compared to the most engaged group (completed 20 bi-weekly surveys); and (iii) a considerable proportion (44.6%) of the participants who stopped completing surveys after 8 weeks continued to share passive Fitbit data for significantly longer (average 42 weeks). Additionally, multivariate survival models showed participants’ age, ownership and brand of smartphones, and recruitment sites to be associated with retention in the study. Together these findings could inform the design of future digital health studies to enable equitable and balanced data collection from diverse populations.

## Introduction

To gain valuable insights into the etiology of depression and identify effective treatments tailored to individuals, large diverse cohorts-based studies are required to assess the underlying temporal patterns in risk and protective factors of depression in individuals^1,2^. However, dynamic day-to-day changes in behavior in naturalistic settings are not captured effectively by conventional clinical assessments that rely on infrequent in-person assessments and subjective retrospective reporting of symptoms^3^. Additionally, reaching and recruiting a large and diverse cohort in a cost-effective and timely manner continues to be challenging for conventional clinical studies^4^.

Due to increasing ubiquity and cost-effectiveness, smartphones and wearable devices, compared to medical devices, allow researchers to monitor personalized daily behaviors and physiology over time for large and diverse populations^5–7^. Combined with scalable data collection platforms, these technologies provide high-fidelity multimodal behavior sensing capabilities^8^. Several recent large-scale remote digital depression studies have shown the feasibility of technology-based remote data collection to assess individuals’ health and behavior^9–12^. For example, sleep^13^, social interactions^14^, and mobility^15–17^ features derived from digital apps, smartphones, or wearable devices, have been demonstrated to be significantly associated with depressive symptoms. Remote digital studies also offer an effective medium to reach and recruit from larger and more diverse populations^18^ thereby considerably lowering the costs and time for creating cohorts of interest than conventional clinical studies^9^.

Although previous remote digital studies have shown the feasibility and utility of leveraging smartphones and wearable technology for assessing behavioral changes in naturalistic settings, long-term participant retention and engagement remain significant challenges^19,20^. Moreover, differential recruitment and retention of participants can lead to imbalanced cohorts and biased data collection that can severely impact the generalizability of findings^21–24^. For example, Pratap et al. found that four specific indicators (referral by clinicians, older age of participants, compensation of participants, and having clinical condition [as opposed to being healthy]) were significantly associated with participant retention, and participant demographics were also associated with long-term engagement patterns in a cross-study evaluation of eight observational digital health studies conducted between 2014–2019^21^.

However, past studies investigated participant behavior and retention in the study for short follow-up periods and were primarily based on active tasks (surveys) completed by participants using a limited set of variables of interest^21–24^. To leverage digital health technology for assessing and managing complex chronic conditions (e.g., psychiatric and neurological disorders), gathering long-term day-to-day behavior change over the long term is necessary. And to remotely engage large populations effectively and equitably, there is a further need to understand key risk factors that impact long-term participant engagement (months to years) in remote digital studies, including the feasibility of collecting active and passive data streams. Participants’ behaviors of answering surveys via the study app, such as time spent responding to surveys and completing surveys in naturalistic settings, may also reflect the participants’ interest in engaging in the study^25–27^. Furthermore, there is a need to understand the feasibility of collecting passive data via smartphones (e.g., Bluetooth and GPS data) and wearables (e.g., heart rate and sleep data) in comparison to active task-based data (e.g., surveys) requiring active participation and with additional user burden.

Here we present findings from a secondary analysis of data collected from the Remote Assessment of Disease and Relapse-Major Depressive Disorder (RADAR-MDD) study^10,28^ to evaluate the potential factors impacting long-term participant retention and engagement in a large, multinational cohort. Specifically, we assessed three specific key questions using participant-level usage data of study apps and wearables: (i) Is participant retention associated with real-world factors (such as sociodemographics, medium of data collection [smartphones and wearables], and severity of depressive symptoms)? (ii) Are there potential patterns in participants’ long-term engagement, including differences between active and passive data streams collected via the study apps and wearables? (iii) And if there are significant differences in participants’ characteristics in the study across different long-term engagement patterns?

## Results

### Cohort characteristics

In total, we analyzed data from 614 participants recruited from three recruitment sites (350, 146, and 118 participants from KCL, CIBER, and VUMC, respectively) between November 2017 to April 2021. The cohort’s median (range) age was 49 (18–80) years; Supplementary Fig. 1 shows the age distribution). The majority of the cohort is females (75.7%, *N* = 465) which is expected because all enrolled participants had a current/prior history of depression, and the prevalence of depression is known to be higher in females than males^29–32^. A subset of 151 (25.1%) participants who were iPhone users were provided with an Android smartphone to use as their primary phone during the study. Differences in participant characteristics across study sites were assessed by Kruskal-Wallis tests^33^. Participants recruited at the CIBER site had the highest median age (54.0 [49.0, 61.0] years) across the three sites (KCL: 45.0 [30.0, 56.0] years and VUMC: 40.0 [26.0, 57.8] years) (*p* < 0.001). In addition, the CIBER site cohort also had a significantly higher median baseline PHQ8 score (15.5 [10.0, 19.0]) than the KCL (9.0 [6.0, 13.0] scores) and VUMC (8.0 [6.0, 14.0] scores) sites (*p* < 0.001). For ethnicity, the majority of recruited participants were white across KCL (84.3%) and VUMC (92.4%) sites. Ethnicity data was not collected for participants recruited at the CIBER site. Table 1 summarizes sociodemographics and clinical characteristics for the overall cohort with comparisons stratified by sites. Briefly, the subcohort with a longer observation period (94 weeks) (See Methods) had 313 participants with a median age of 51.0 [37.0, 59.0] years, with the majority being females (75.1%, *N* = 235). The full set of secondary cohort descriptive statistics is summarized in Supplementary Table 1.

**Table 1:** A summary of characteristics of 614 participants in the RADAR-MDD study, with comparisons across the three study sites using Kruskal-Wallis tests.

Characteristics	Total	KCL	CIBER	VUMC	*p* value
Number of participants, *n*	614	350	146	118	
Age (median [IQR])	49.00 [32.00, 58.75]	45.00 [30.00, 56.00]	54.00 [49.00, 61.00]	40.00 [26.00, 57.75]	<0.001
Female, *n* (%)	465 (75.7)	267 (76.3)	106 (72.6)	92 (78.0)	0.56
Marital status, *n* (%)					0.005
Single/separated/divorced/widowed	328(53.4)	185(52.9)	66 (45.2)	77 (65.3)	
Married/cohabiting/LTR	286 (46.6)	165 (47.1)	80 (54.8)	41 (34.8)	
Ethnicity, *n* (%)					<0.001
White	404 (86.3)	295 (84.3)	–	109 (92.4)	
Black	14 (3.0)	11 (3.1)	–	3 (2.5)	
Asian	16 (3.4)	16 (4.6)	–	0 (0)	
Other	34 (7.3)	28 (8.0)	–	6 (5.1)	
Employed, *n* (%)	258 (42.0)	186 (53.1)	33 (22.6)	39 (33.1)	<0.001
Having children, *n* (%)	304(49.5)	152 (43.4)	111 (76.0)	41 (34.8)	<0.001
Years in education (median [IQR])	16.00 [13.00, 19.00]	17.00 [14.00, 19.00]	11.00 [9.00, 15.75]	16.50 [14.00, 20.00]	<0.001
Annual income, *n* (%)					<0.001
<15,000 (£/€)	152 (24.8)	74 (21.1)	47 (32.2)	31 (26.3)	
15,000–55,000 (£/€)	348 (56.7)	203 (58.0)	92 (63.0)	53 (44.9)	
55,000 (£/€)	98 (16.0)	72 (20.6)	7 (4.8)	19 (16.1)	
Accommodation, *n* (%)					<0.001
Own outright/with mortgage	323 (52.6)	169 (48.3)	105 (71.9)	49 (41.5)	
Renting	236 (38.4)	151 (43.1)	27 (18.5)	58 (49.2)	
Living rent-free	46 (7.5)	29 (8.3)	10 (6.8)	7 (5.9)	
Baseline PHQ8 score (median [IQR])	10.00 [7.00, 16.00]	9.00 [6.00, 13.00]	15.50 [10.00, 19.00]	8.00 [6.00, 14.00]	<0.001
Having comorbidities, *n* (%)	311 (50.7)	176 (50.3)	96 (65.8)	39 (33.1)	
Taking depression medication, *n* (%)	400 (65.1)	206 (58.9)	133 (91.1)	61 (51.7)	
Number of contact logs (median [IQR])	4.00 [2.00, 7.00]	5.00 [3.00, 8.00]	3.00 [2.00, 5.00]	2.00 [1.00, 3.75]	<0.001
Provided phone, *n* (%)	151 (25.1)	120 (34.9)	8 (5.7)	23 (19.5)	<0.001
Brand of smartphone, *n* (%)					<0.001
Motorola	240 (39.7)	171 (49.7)	30 (20.8)	39 (33.3)	
Samsung	194 (32.1)	94 (27.3)	44 (30.6)	56 (47.9)	
Other	171 (28.3)	79 (23.0)	70 (48.6)	22 (18.8)	

Note, ethnicity data was not collected for participants recruited at the CIBER site.

### Participant retention

**Fig. 1:**
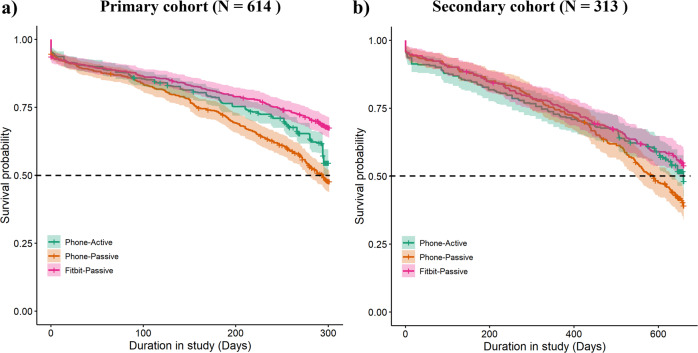
Participant retention in the RADAR-MDD study. The Kaplan-Meier survival curves for (**a**) the primary cohort (*N* = 614) with an observation period of 43 weeks, and (**b**) the secondary cohort (*N* = 313) with a longer observation period of 94 weeks stratified by Phone-Active, Phone-Passive, and Fitbit-Passive data streams.

For the primary cohort analysis, the participant retention (survival rate) at the end of the common maximum observation period of 43 weeks (described in Methods) as quantified using Phone-Active, Phone-Passive, and Fitbit-Passive data streams were 54.6% (*N* = 335), 47.7% (*N* = 293), and 67.6% (*N* = 415), respectively. Similarly, for the secondary cohort, the participant retention rates in the 94 weeks measured by the three data streams were 48.2% (*N* = 151), 39.3% (*N* = 123), and 54.0% (*N* = 169), respectively. Figure 1 displays the Kaplan-Meier survival curves that show participant retention across two observation periods stratified by three data streams.

To further assess the impact of multiple variables of interest (age, gender, marital status, employment, children, education, income, accommodation, the baseline PHQ8 score, comorbidity, depression medication, phone status, smartphone brand, and study site), we used three multivariate Cox Proportional-Hazards models^34^ for Phone-Active, Phone-Passive, and Fitbit-Passive data streams, respectively. All variables, except gender variable in the Phone-Passive model, met the proportional hazards assumption. We added a time interaction term to the gender variable to meet the CoxPH model assumptions^35,36^. See Supplementary Table 2 for the results of proportional hazard assumption tests and Supplementary Figs. 2–4 for the scaled Schoenfeld residuals plots. Table 2 shows hazard ratio (HR) estimates and 95% confidence intervals of all variables of the three models.

**Table 2:** The estimates and 95% confidence intervals of hazard ratio (HR) of variables in the Cox models for assessing the impact of multiple variables of interest on the participant retention time in the first 43 weeks of the study for Phone-Active, Phone-Passive, and Fitbit-Passive data streams, respectively.

Variables		Phone-Active		Phone-Passive		Fitbit-Passive	
		HR (95% CI)	*p*	HR (95% CI)	*p*	HR (95% CI)	*p*
Age	<30	Reference	–	Reference	–	Reference	–
	30–39	0.8 (0.53–1.2)	0.28	0.81 (0.54–1.21)	0.30	0.7 (0.4–1.22)	0.21
	40–49	0.75 (0.48–1.17)	0.20	0.76 (0.49–1.19)	0.23	0.5 (0.27–0.93)	0.03
	50–59	0.73 (0.46–1.15)	0.18	0.74 (0.47–1.17)	0.20	0.59 (0.33–1.08)	0.09
	>60	0.56 (0.34–0.93)	0.02	0.56 (0.34–0.93)	0.02	0.42 (0.22–0.81)	0.01
Gender	Female	Reference	–	–	–	Reference	–
	Male	0.85 (0.63–1.15)	0.29	–	–	0.79 (0.54–1.18)	0.25
Marital status	Single	Reference	–	Reference	–	Reference	–
	Married	0.97 (0.72–1.31)	0.85	0.96 (0.71–1.29)	0.78	0.84 (0.58–1.23)	0.37
Employment	No	Reference	–	Reference	–	Reference	–
	Yes	0.86 (0.63–1.16)	0.32	0.85 (0.63–1.15)	0.28	0.75 (0.5–1.11)	0.14
Having children	No	Reference	–	Reference	–	Reference	–
	Yes	0.93 (0.66–1.31)	0.68	0.94 (0.67–1.32)	0.71	1.45 (0.93–2.26)	0.10
Years in education		1.01 (0.99–1.03)	0.19	1.01 (0.99–1.03)	0.21	0.99 (0.96–1.02)	0.49
Annual income (£/€)	<15,000	Reference	–	Reference	–	Reference	–
	15,000–55,000	1.13 (0.82–1.57)	0.46	1.14 (0.82–1.59)	0.43	0.95 (0.64–1.42)	0.81
	>55,000	0.98 (0.6–1.61)	0.95	1 (0.61–1.65)	0.99	0.75 (0.38–1.47)	0.40
Accommodation	Own outright	Reference	–	Reference	–	Reference	–
	Renting	1.14 (0.84–1.54)	0.40	1.14 (0.84–1.54)	0.41	0.84 (0.57–1.24)	0.38
	Living rent-free	0.8 (0.46–1.39)	0.42	0.79 (0.45–1.38)	0.41	0.59 (0.29–1.21)	0.15
PHQ8 score		1.01 (0.98–1.03)	0.59	1.01 (0.99–1.03)	0.50	1 (0.97–1.03)	0.81
Having comorbidities	No	Reference	–	Reference	–	Reference	–
	Yes	0.88 (0.68–1.16)	0.37	0.88 (0.67–1.15)	0.33	1.17 (0.82–1.66)	0.39
Medication	No	Reference	–	Reference	–	Reference	–
	Yes	1.14 (0.86–1.51)	0.36	1.13 (0.86–1.5)	0.38	1.06 (0.72–1.55)	0.77
Study site	CIBER	Reference	–	Reference	–	Reference	–
	KCL	0.86 (0.59–1.26)	0.43	0.87 (0.59–1.27)	0.47	0.59 (0.38–0.94)	0.03
	VUMC	1.49 (0.95–2.34)	0.09	1.54 (0.98–2.43)	0.06	0.4 (0.21–0.76)	<0.001
Brand of smartphone	Other	Reference	–	Reference	–	Reference	–
	Motorola	0.26 (0.17–0.4)	<0.001	0.26 (0.17–0.4)	<0.001	0.56 (0.34–0.92)	0.02
	Samsung	0.58 (0.43–0.79)	<0.001	0.57 (0.42–0.77)	<0.001	0.78 (0.53–1.16)	0.22
Phone status	Own phone	Reference	–	Reference	–	Reference	–
	Provided phone	1.67 (1.06–2.64)	0.03	1.65 (1.05–2.6)	0.03	1.61 (0.93–2.77)	0.09

Note, in the Phone-Passive model, an interaction term of the “gender” variable with a split time variable (cut points are 130 and 240) was used to make the variable meet the model assumption. HR of Male (1–129) = 0.67 (0.39–1.14) (*p* = 0.14), HR of Male (130–239) = 0.58 (0.34–0.99) (*p* = 0.05), and HR of Male (240–301) = 1.52 (0.95–2.42) (*p* = 0.08).

For each predictor, a HR estimate greater than 1 indicates the variable is associated with a higher risk of participants not contributing data to the study thus negatively impacting participant retention in the study. Across the three data streams, age was found to significantly affect participant retention in the study. Compared with the youngest group (18–30 years old), participants in older age groups tend to stay in the study for a longer time. Participants in the oldest group (>60 years old) had the lowest risks of stopping contributing data for all three data streams (Phone-Active: HR = 0.56, *p* = 0.02; Phone-Passive: HR = 0.56, *p* = 0.02; Fitbit-Passive: HR = 0.42, *p* = 0.01). Compared to participants with their own smartphones, those using the study provided Android phone had a statistically significantly higher risk for not contributing phone data actively (HR = 1.67, *p* = 0.03) and passively (HR = 1.65, *p* = 0.03). Of note, participants using Motorola (HR = 0.26, *p* < 0.001) and Samsung (HR = 0.57–0.58, *p* < 0.001) branded phones also contributed both active and passive phone data for significantly longer durations compared with other brands of smartphones. Furthermore, compared with the CIBER site, participants in the KCL and VUMC sites had the lower risk of stopping sharing the Fitbit-Passive data (KCL: HR = 0.59, *p* = 0.03, VUMC: HR = 0.40, *p* < 0.001).

Participants’ age also continued to significantly impact retention in the extended observation period (94 weeks) across all three data streams assessed in the secondary cohort (Supplementary Table 3).

### Participants’ long-term engagement patterns in the study

**Fig. 2:**
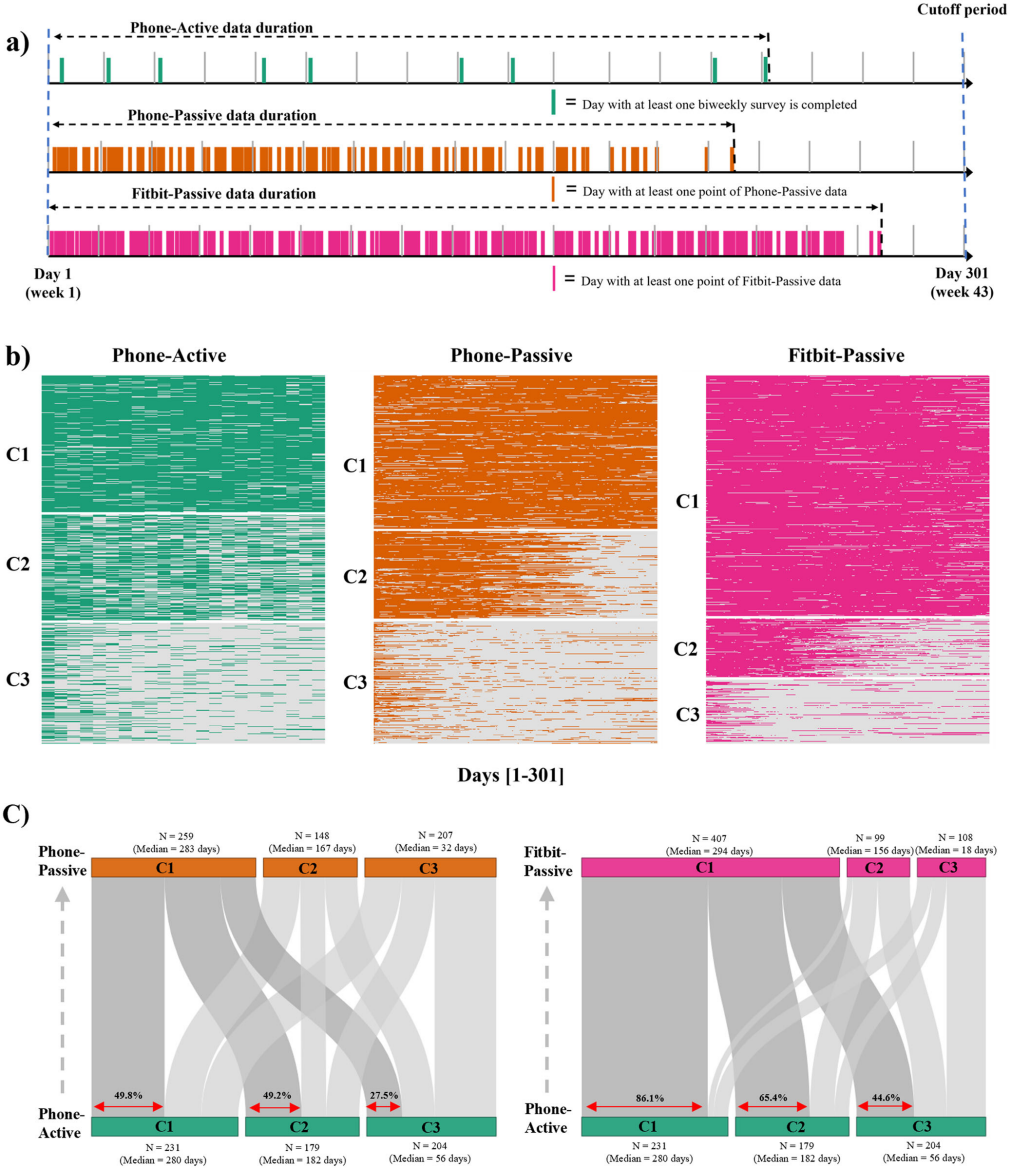
Participant long-term engagement patterns in the RADAR-MDD dataset. **a** Schematic representation of a participant’s 3 data streams in the study. **b** Heatmaps of participant longitudinal engagement patterns, clustered using K-means clustering. In each heatmap, each row represents a data-availability vector of one participant (described in Methods), and subgroups were arranged from the most engaged cluster to the least engaged cluster (C1-C3). **c** Sankey plots showing the proportion of common participants between clusters determined from Phone-Active (green), Phone-Passive (brown), and Fitbit-Passive (pink) data streams. To match passive data streams, if a survey that was due every two weeks was completed by a participant, 14 elements of the participant’s data-availability vector of Phone-Active corresponding to these two weeks are set to 1 (representing the participant was contributing active data) (See “Methods” for further details).

**Fig. 3:**
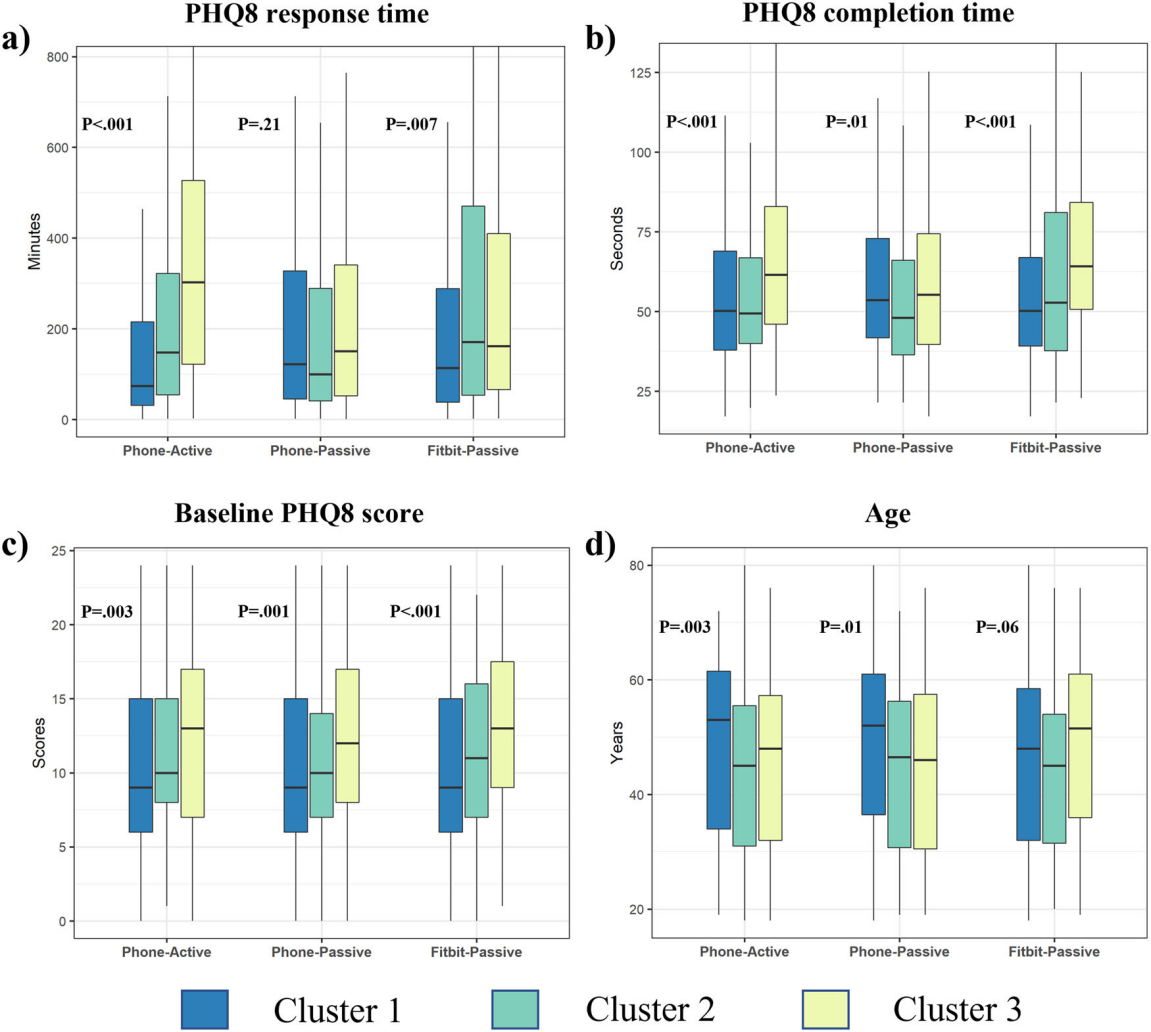
Significant factors impacting the long-term engagement patterns. Significant differences in participants’ (**a**) survey response time, (**b**) survey completion time, (**c**) baseline depression symptom severity, and (**d**) age across three long-term engagement patterns (Cluster 1, Cluster 2, and Cluster 3) for Phone-Active, Phone-Passive, and Fitbit-Passive data streams, respectively. Note: Cluster 1, Cluster 2, and Cluster 3 represent the most engaged, medium engaged, and least engaged patterns shown in Fig. 2b. The centerline of the boxplots shows the median value of the factor across participants for each cluster, the box indicates the interquartile range (IQR) from the 25th (Q1) to 75th (Q3) percentiles, and the whiskers represent points within Q1 − 1.5*IQR and Q3 + 1.5*IQR. All p values were calculated by Kruskal-Wallis tests.

Patterns in participants’ day-to-day data sharing were assessed using an unsupervised K-means method^37^ across the three data streams separately (Fig. 2a). In the primary observation period, three subgroups showing distinct participant engagement patterns (C1: most engaged, C2: medium engaged, and C3: least engaged), emerged across each data stream (Fig. 2b). Across the three engagement clusters in each data stream (Phone-Active, Phone-Passive, and Fitbit-Passive), we found notable differences in participants’ behavior (survey response and completion times), baseline depression symptom severity, and age (Fig. 3). Supplementary Tables 5–7 provide further details for comparisons of all variables across three clusters for all three data streams using the Kruskal-Wallis tests.

#### Long-term engagement patterns across phone active, phone passive, and wearable data streams

Participants in the most engaged C1 cluster (37.6% of the cohort; *N* = 231) completed a median (IQR) of 20.0 (18.0, 21.0) bi-weekly surveys as opposed to 4.0 (1.0, 6.0) for those in the least engaged cluster (C3; 33.2% of the cohort; *N* = 204). Similarly, the data sharing patterns for passive data streams showed significant differences. Participants (42.2% of the cohort; *N* = 259) in the most engaged C1 cluster of the Phone-Passive data stream, shared phone-based passive data for a median (IQR) of 283 (257.0, 298.0) days as opposed to 32 (4.0, 67.5) days for the participants in the least engaged C3 cluster (33.7%; *N* = 207). Similarly, for the Fitbit-based data gathered passively, the most engaged C1 cluster with 66.3% participants (*N* = 407) shared the data for median (IQR) 294 (274.0, 301.0) days compared to just 18 (0, 67.0) days for the 17.6% participants (*N* = 108) in the least engaged cluster (C3). Of note, we found a considerable proportion of participants in the medium (C2) and least (C3) engaged clusters of the Phone-Active data stream, despite completing a lesser number of active surveys (13 and 4 bi-weekly surveys, respectively), continued contributing passive data from Fitbit for an average of 42 weeks. Figure 2c shows this marked transition where 65.4% of participants (*N* = 151) from the C2 cluster and 44.6% of participants (*N* = 91) from the C3 cluster, based on the Phone-Active data stream, transitioned to the most engaged C1 cluster of the Fitbit-Passive data stream.

#### Survey response and completion times

We also observed prominent linkages between long-term engagement and the survey response time (the time to respond to survey notifications) and completion time (total survey completion time). Participants in the most engaged C1 cluster of the Phone-Active data stream had significantly shorter survey response time (73.7 [31.3, 215.8] minutes) for the PHQ8 survey compared to 302.4 (122.3, 527.1) minutes for the least engaged C3 cluster (Fig. 3a) (*p* < 0.001). This finding is also consistent for subgroups in the Fitbit-Passive data stream (Fig. 3a) and RSES survey (Supplementary Tables 5, 7). In terms of survey completion time, participants in the least engaged cluster (C3) of the Phone-Active data stream took significantly longer (61.6 [46.1, 83.0] seconds) to complete surveys than those in C1 (50.3 [37.9, 69.0] seconds) and C2 (49.4 [40.0, 67.0] seconds) clusters (Fig. 3b) (*p* < 0.001). Likewise, the finding of survey completion time is consistent for the Fitbit-Passive data stream (Fig. 3b) and RSES survey (Supplementary Tables 5, 7).

#### Baseline depression symptom severity

The baseline PHQ8 scores of participants were significantly different across three subgroups (C1, C2, C3) for all three data streams. Overall, participants in the least engaged cluster (C3) had significantly higher severity of depressive symptoms at enrollment (Fig. 3c). For example, participants in C3 for the Phone-Active data stream had a 4 points difference in the median baseline PHQ8 score (13.0 [7.0, 17.0]) compared to participants in the most engaged cluster (C1) with a median baseline PHQ8 score of 9.0 (6.0, 15.0) (*p* = 0.003). Similarly, in participants in cluster C3 of Phone-Passive and Fitbit-Passive data streams showed a statistically significant difference in the baseline PHQ8 scores compared with the most engaged cluster (C1) (Phone-Passive - C1: 9 [6.0, 15.0]; C3: 12 [8.0,17.0] and Fitbit-Passive - C1: 9 [6.0, 15.0]; C3: 13 [9.0, 17.5]) (*p* < 0.001).

#### Sociodemographics

The age of participants was significantly different across the 3 clusters of Phone-Active and Phone-Passive data streams. For the Phone-Active data stream, participants in C1 cluster had a significantly higher median (IQR) age of 53.0 (34.0, 61.5) years than participants in C2 (45.0 [31.0, 55.5]) and C3 (48.0 [32.0, 57.3]) clusters (*p* = 0.003). Similarly, for the Phone-Passive data stream, participants in the most active C1 cluster had the significantly highest median (IQR) age of 52.0 (36.5, 61.0) years across the 3 clusters (C2: 46.5 [30.8, 56.3] years and C3: 46.0 [30.5, 57.5] years) (*p* = 0.01). For ethnicity (available for KCL and VUMC sites), we found the proportion of white participants was significantly lower in the least engaged C3 group (77.8%) than C1 (95.1%) and C2 (84.0%) clusters for the Phone-Active data (*p* < 0.001). Likewise, Phone-Passive and Fitbit-Passive data had similar findings (Supplementary Table 11).

#### Phone brand, phone status, and “human-in-the-loop” (research team contacting participants)

We found the Phone-Passive data collection to be significantly different across the smartphone brands. In the Phone-Passive data stream, the proportion of participants with Motorola brand phones in the least engaged C3 cluster (15%) was significantly lower than C1 (57.0%) and C2 (42.9%) (*p* < 0.001) (Supplementary Table 6). Also, the proportion of participants using study provided phones in the C3 cluster (11.7%) was significantly lower than C1 (32.6%) and C2 (29.9%) clusters (*p* < 0.001) (Supplementary Table 6). Further, for Phone-Active data stream, we found participants in the most engaged C1 cluster were contacted less frequently (3.0 [2.0, 5.0]) than those in the C2 (5.0 [3.0, 7.0]) and C3 (5.0 [2.0, 9.0]) clusters (*p* < 0.001) (Supplementary Table 5).

For the secondary cohort with a longer observation period, unsupervised clustering of 94 weeks of individual-level engagement data showed 4 clusters (C1–C4) shown in Supplementary Fig. 6. Results of the participant characteristics enriched in the 4 engagement clusters for the secondary cohort are similar to the results of the primary cohort and are summarized in Supplementary Tables 8–10 for the three data streams, respectively.

## Discussion

We report findings regarding long-term participant retention and engagement patterns from a large European multinational remote digital study for depression^10,28^. Our findings show a significantly higher long-term participant retention than in past remote digital health studies^19,21–24^. However, we show several factors, that can significantly impact long-term participant retention and the density of data collection in naturalistic settings. These range from participants’ sociodemographics, and depression symptom severity, to study app usage behavior e.g., survey response and completion times. Here we contextualize our key findings in the broader digital medicine context that may help inform the design and development of remote digital studies. We also compare the utility of using active and passive data collection for long-term remote monitoring of behavior and health outcomes. Finally, we share some of the participant engagement strategies deployed by the RADAR-MDD consortium^10^ and data-driven insights to help improve long-term participant engagement in future remote digital studies.

One of the notable findings was that participants with higher severity of depression at the time of enrollment contributed less data both actively and passively. For example, participants in the least engaged cluster (C3) had the highest depression severity at the baseline and were up to 16 times less likely to share active or passive data from smartphones and wearables. The finding indicates that participants with higher depression symptom severity may be at a higher risk of not engaging in fully remote studies. A similar finding that the lowest engaged group had the highest depression and anxiety scores was observed in a previous web-based mental health intervention study^38^. Non-uniform engagement in depression study apps, particularly by participants with higher depression severity could bias the data collection impacting the generalizability and robustness of generated evidence. There is an urgent need for future research to develop solutions that alleviate non-uniform data collection. First, mixed methods research that aims to uncover the context behind quantitative findings by using qualitative methods^39^ is needed to understand issues that impact the engagement of people with high depression severity. Second, co-designing study protocols and apps with representative patient advisory boards can help optimize the acceptability of the technology^40,41^. Third, applying “Human-in-the-loop”^42,43^ approaches can help the timely resolution of problems that are encountered by participants and may reduce the risk of disengaging from the study. Finally, the present study showed that passive data gathered from wearables has greater contiguity and participant retention over the long term. Focusing efforts on collecting multimodal passive data streams without additional user burden may be a more effective and acceptable marker of individual behavior in naturalistic settings^44^. We discuss these strategies in further detail below.

We also observed participants’ time in responding to and completing surveys is significantly associated with their long-term engagement patterns. Participants with shorter survey response and completion times tend to engage for the longer term completing more surveys and wearing Fitbit for a significantly longer period. Past studies have also reported that if participants are more interested in the study, they are quicker to respond and complete study-related assessments^25–27^. Further, survey response and completion time may also be correlated with several other factors, such as participants’ familiarity with smartphones and study apps, life behaviors, and smartphone latency (battery and memory). Such objective metrics on participants’ app-usage behavior may be potentially useful for passively assessing the quality of the active data and predicting long-term engagement early.

In addition, nearly a quarter of participants were provided an Android smartphone by the research team to be used as their primary phone during the study observation period. However, the study engagement data shows that participants using study provided phones were more likely to stop sharing their phone data (both active and passive data) compared with other participants who used their own smartphones. One possible reason for reduced engagement may be linked to participants not using the provided phones as their primary phones in daily life.

Finally, we found that age is a significant indicator of participant retention and engagement. Older participants have a lower risk of disengaging from the study app (Table 2) and tend to contribute more surveys and phone passive data (Fig. 3d) than the younger participants. This finding is consistent with several previous engagement studies.^21,45,46^

This study also demonstrated the feasibility of collecting active and passive data streams for long-term behavior monitoring. While there is growing interest amongst researchers in gathering behavioral data without having to rely on episodic in-clinic assessments that may be subject to recall bias^47^, there is limited empirical research quantifying the long-term participant engagement differences between active (surveys) and passive data streams (smartphones and wearables). We compared the long-term differences in the density of active and passive data collected from surveys, smartphones, and wearable devices.

Passively gathered data from wearable devices showed the highest long-term engagement (C1 in Fig. 2b and Supplementary Fig. 6) and the highest participant retention rates (Fig. 1) over both observation periods (43 weeks and 94 weeks). The finding clearly shows that wearable devices with minimal participant burden could help researchers collect high-density data over the longer term. Another potential reason may be that the Fitbit app provides participants with timely feedback about their sleep quality and physical activity, which may increase their interest in wearing Fitbit devices. We found a significant proportion of the participants who completed fewer longitudinal surveys (C2 and C3 of the Phone-Active data stream) but contributed passive Fitbit data for significantly longer (Fig. 2c). This illustrates the value of wearable devices for long-term monitoring of participants who cannot routinely actively engage in completing frequent health surveys.

On the other hand, we found that the passive data gathered from participants’ phones had the lowest retention rate in both observation periods (Fig. 1). A potential reason for lower compliance in passive data collection from smartphones could be due to the relatively high consumption of battery and users’ data plan. The study app collected high-resolution passive data frequently (e.g., GPS [every 10 min], Bluetooth [hourly], battery levels [every 10 min], and phone usage [event trigger]). The collection of highly granular passive data could have made some participants stop the app from collecting passive data or uninstall it. Future research is needed to understand the suitable balance between passive data collection and phone battery consumption acceptable to participants in their daily lives. Notably, we also found that smartphone brands significantly affected the retention and density of phone data collection. Smartphone brands may have different policies on the duration for which an app can collect granular passive data continuously. However, the sample sizes of several categories of phone brands were limited in our cohort. Additional research is needed to investigate intra-device/brand differences within and across Android and iOS phones to enable the robust and equitable collection of passive data. Finally, a small but significant group of participants were not contributing either active or passive data (Fig. 2c). Further research is needed to understand the concerns of this subgroup to avoid the collection of unbalanced data.

We discuss four strategies developed and adopted by the RADAR-MDD consortium^10^ which may have helped increase long-term participant retention and engagement in the RADAR-MDD study.

### “Human-in-the-loop”^43^

The RADAR-MDD research team contacted participants for various reasons, such as reminding 3-month assessments, any malfunction in the Fitbit device, problems in study apps, and congratulating participants for completing the 1-year milestone. Timely resolution of technical issues and feedback and encouragement from the research team may help keep participants in the study^22^.

### Monetary incentives

Compensation for participant time and monetary incentives are known to enhance engagement^22,48^. Although participants were not offered compensation for completing surveys remotely and sharing behavior data passively, existing monetary incentives could increase participants’ willingness to remain engaged in the study. For example, participants were given monetary incentives for enrolling in the study, taking part in clinical assessments (every 3 months), and additional interviews (e.g., 1-year interview) (see “Methods” section). This cyclical compensation (every 3 months and 1 year) could have indirectly incentivized participants to remain in the study. Furthermore, participants were allowed to keep the Fitbit device after the completion of the study which could have impacted their motivation to join and remain engaged in the study for a longer term.

### Participant-centric design

Participants’ lack of familiarity with how to use digital technologies (study apps) and lack of intrinsic motivation (not familiar with the value of the study) are two key barriers to long-term engagement^22^. Therefore, participants and patients were invited to provide input at all stages of the study process^10^. A patient advisory board comprising service users guided the early study protocol and study app design stages to the implementation and analysis phases. They contributed to improving the study design and engagement motivation strategy and shaped how the technology was used^49^. This approach, called “participant-centered initiative”^50,51^, treats participants as partners in the entire research cycle, which could provide a means to improve participant retention and engagement in long-term digital health studies. A recent study demonstrated that the “participant-centric design” played an essential role in maximizing engagement in remote app-based studies^19^.

### Recruiting participants with the target disease of interest

The inclusion criteria in the present study required all participants to have at least one depressive episode in the last two years. Therefore, the study contains an enriched population with a specific clinical condition. Prior research has shown that participants with clinical conditions of interest in the study tend to remain engaged for significantly longer^21,22^. Experiences of having depression may make participants aware of the benefits of regularly completing the self-assessment and getting feedback from clinical teams to realize their status of mental health^22^.

Although the incentives and recruitment strategies discussed above increased participant retention in the present cohort, a notable proportion of the cohort (17.59–33.71%) across active and passive data streams did not remain engaged in the study over the long-term (C3 clusters in Fig. 2b). Long-term participant retention and engagement in remote digital studies, therefore, remains an active area of research. Several potential solutions could be learned from our findings. Participant characteristics, such as younger age, more depressive symptoms at baseline, and delayed responses to remote surveys, could act as early indicators of a subgroup of participants at a higher risk of disengagement from the study. Targeted engagement strategies including tailored communication and increased “Human-in-loop” interactions could be deployed to this subgroup. An alternative approach is to recruit more heavily from participants matching the characteristics of the low engagers, which may help reduce the overall data imbalance.

Also, a near-real-time analytical framework could be deployed to monitor the incoming data for known socio-technical biases continually. The system could triage participants who are falling below an acceptable level of compliance to the study team in terms of data quantity and quality. This could help just-in-time identify potential causes of unbalanced data collection and allow for timely and targeted interventions to re-engage participants at the highest risk of drop.

Our findings should be viewed in the context of certain limitations related to the data collection in a fully remote European multinational remote digital study for depression. First, the RADAR-MDD study used an open enrollment model to gather data and did not stratify or randomize participant recruitment based on sociodemographic characteristics, enrollment sites, etc. For example, the overall cohort had significantly fewer participants older than 70 years, which can be related to known barriers e.g., lower use of digital technologies and health problems^52–54^. Further, the study population was predominantly white people with the majority of females. While the higher proportion of females in the present study cohort is aligned with previous epidemiological and remote observational studies^55–57^ and a known higher prevalence of depression in females than in males^29–32^; the findings may not be generalizable to a more diverse or non-depressed population. Future studies should use randomized designs to investigate the causal impact of various demographic and sociotechnical factors on participant retention using a representative target population linked to the condition of interest.

Second, the depression symptom severity in the study cohort was measured using the PHQ8 survey. Although the PHQ survey is a viable indicator of depression severity^58–60^ and has been used in a number of remote studies^20,61,62^, the PHQ survey was designed for the rapid depression screen and not suggested for depression diagnosis^63,64^. Third, there were some changes during the course of the study such as changes in versions of some surveys, fixing technical bugs (e.g., missing notifications), and addition of surveys made as well as different study start times across three sites that could impact participant engagement. Fourth, the education system, language, income levels, and currency are also different across European countries and could lead to inconsistencies in the comparison of participant responses to socio-demographic questions across sites. These potential differences limited our interpretation of the different levels of participant engagement across three sites. Fifth, the technical differences between the two versions of Fitbit devices (Charge 2 and Charge 3) deployed in the study were not tracked. Also, the present study was only based on Android smartphone operating system. As a result, the impact due to different versions of wearable devices and different smartphone operating systems on participant engagement is unclear.

Sixth, the specific impact of the number of contact logs on participant engagement may be bidirectional. For example, technical issues may decrease participant engagement with the study app despite the study team reaching out. On the other hand, reaching out to remind participants to complete an assessment or congratulating them for reaching the 1-year milestone may increase participant engagement. Also, participants in the most engaged clusters had the lowest number of contact logs, indicating that highly engaged participants did not need additional reminders to complete assessments, and encountered fewer technical issues.

Seventh, while participants were not paid for completing remote surveys via smartphones or sharing passive data, compensation was given for clinical assessments every 3 months, which may also affect the generalizability of our findings in cohorts without any incentives.

This study demonstrated that participant retention in the RADAR-MDD study was significantly higher than in past digital studies. Higher retention is likely linked to the deployment of several engagement strategies such as “human-in-the-loop”, monetary incentives, participant-centric design, and a targeted clinical cohort. We found several notable indicators such as age, depression severity, and survey response and competition times in the study app significantly impacted the depth and density of our data collection in fully remote research. Furthermore, passive data gathered from wearables without participant burden showed advantages in helping collect behavioral data with greater contiguity and over a longer duration. Combined, these objective engagement metrics could help identify and triage participants with the highest dropout risk to tailored and just-in-time engagement to enable equitable and balanced health data collection from diverse target populations.

## Methods

### RADAR-MDD study design

Data used in this study was collected from the EU research program RADAR-MDD, which aimed to investigate the utility of smartphones and wearable devices to monitor depression remotely and understand factors that could help predict relapse in major depressive disorder^10^. The study recruited 623 participants from 3 sites across 3 Europe countries (United Kingdom - King’s College London [KCL]; Spain - Centro de Investigación Biomédican en Red [CIBER]; Netherlands - Vrije Universiteit Medisch Centrum [VUMC]) and followed participants for up to 2 years^10^. Nine participants recruited from a second site in Spain were not included in the present analysis due to the small sample size. All participants in this study were above 18 years old and had a history of recurrent MDD with at least one episode within the last 2 years that meets DSM-5 diagnostic criteria for diagnosis of MDD. Additionally, in order to be enrolled in the study, the participants were asked to use an Android smartphone as their primary phone if they had one or were provided with one to use if they did not.

The study used RADAR-base, an open-source platform, for smartphone-based health data collection via two Android study apps (active and passive monitoring apps)^8^. Participants were asked to regularly complete self-reported surveys via the active app^10^. Additionally, participants’ daily behavior was gathered passively using the Android passive monitoring app and a Fitbit wearable (details below). The participants were also required to complete some clinical assessments via research electronic data capture (REDCap) surveys every 3 months. Participants’ socio-demographics, medical history, lifetime depression history, and baseline mental health status were also collected during the participant enrollment session^10^. Although participants were not financially reimbursed for providing data via study apps and the wearable, participants received £15/€20 for enrollment, £5/€10 for clinical assessments (REDCap surveys) every 3 months, and £10/€10 for every additional qualitative interview completed^10^. Furthermore, the “Human-in-the-loop”^43^ approach was used during the observation period. The research team contacted participants for various reasons, such as reminding clinical assessments, technical issues (e.g., Fitbit broken, problems in study apps, and phone issues), and congratulating participants on reaching key study milestones (e.g., one year in the study). The detailed study protocol and descriptions of the dataset have been reported by Matcham et al.^10,28^.

The first participant was enrolled in November 2017 and the last participant was enrolled in June 2020, and the data collection was finished in April 2021^28^. As a result of this rolling enrollment, the time in study for RADAR-MDD participants varies from 11 months to 24 months. There were temporal differences in participant recruitment across the three sites. The KCL site started participant recruitment first (November 2017) followed by the CIBER site (September 2018), and the VUMC enrolled participants later again (February 2019)^28^.

The RADAR-MDD protocol was co-developed together with a patient advisory board who shared their opinions on several user-facing aspects of the study including the choice and frequency of survey measures, the usability of the study app, participant-facing documents, selection of optimal participation incentives, selection and deployment of wearable device as well as the data analysis plan^10,65^. All participants signed informed consent and the study had been approved by all local Ethics committees^10^.

RADAR-MDD was conducted per the Declaration of Helsinki and Good Clinical Practice, adhering to principles outlined in the NHS Research Governance Framework for Health and Social Care (2nd edition). Ethical approval has been obtained in London from the Camberwell St Giles Research Ethics Committee (REC reference: 17/LO/1154), in Spain from the CEIC Fundacio Sant Joan de Deu (CI: PIC-128–17) and in The Netherlands from the Medische Ethische Toetsingscommissie VUmc (METc VUmc registratienummer: 2018.012 – NL63557.029.17)

### Primary data streams

For evaluating long-term participant retention and engagement in the study, we classified the data collected by the study apps into three distinct categories: (i) Phone active data - representing active tasks completed by participants via the study app, (ii) Phone passive data - continuous data streams gathered by the smartphones without active input from participants, and (iii) Fitbit passive data - continual physiological monitoring data collected through a wrist-worn Fitbit device during the observation period.

### Phone active data

A variety of episodic surveys were administered via the study app. The complete list of surveys and deployment details are covered in the study protocol^10^. However, with the focus on present research evaluating long-term engagement, we considered the two longitudinal surveys, the 8-item Patient Health Questionnaire (PHQ8^66^) and Rosenberg Self-Esteem Scale (RSES^67^), which were conducted via smartphones remotely once every two weeks. The completion windows for PHQ8 and RSES are both 3 days. Surveys could not be completed once the window expired. If the participants finished at least one of these two surveys, we considered they were engaging in the active assessments part of the study for the corresponding 2 weeks.

### Phone passive data

The passive monitoring app unobtrusively and continuously collected information on participants’ phone usage (e.g., battery level logs, app use logs, and phone interaction data) and surrounding information (e.g., ambient light, nearby Bluetooth device count, and GPS location data)^10^. We considered a participant to be using their study phone and sharing the phone passive data on a given day if at least one passive data point was collected from their smartphone during the day.

### Fitbit passive data

Participants were also required to wear a Fitbit Charge 2 or 3 wrist-worn during the follow-up time to provide passive measures of their sleep stages, steps, calorie consumption, and heart rate. Similarly, if at least one data point from the Fitbit-based data stream was available, we considered the participant to be wearing the Fitbit at least once during that given day.

### Primary outcomes

We defined two key metrics to assess the participant’s engagement. (i) Duration in the study: the number of days between the first and last day of data contributed by the participant in a selected engagement observation period. (ii) Longitudinal data-availability vector: a binary-encoded vector representing the density of the participant’s contributed data in an engagement observation period, where the i-th element of the vector represents the i-th day in the study and is set to 1 if a data point is contributed by the participant on that day or is set to 0 otherwise. To align the frequency of passive data streams (daily), for the Phone-Active data, we set the 14 elements (2-week period) of the data-availability vector to 1 in which a survey was completed by a participant. We calculated these two metrics of engagement for each of the three data streams (Phone-Active, Phone-Passive, and Fitbit-Passive data), respectively.

### Variables of interest

A variety of factors may affect the duration and density of participants’ engagement in remote digital studies^21,68,69^. In this engagement study, we considered a variety of factors including participants’ socio-demographics, the study site, the smartphone brand, baseline depression symptom severity, comorbidity, depression medication, as well as app usage behavior (survey response time and survey completion time) as variables of interest. These are briefly described below.

#### Sociodemographics

Age, gender, ethnicity (not collected at the CIBER site), education, marital status, income, and accommodation type, were recorded in the enrollment session.

#### Study site

Participant recruitment site (KCL, CIBER, and VUMC).

#### Phone status

Participants who did not have an Android smartphone were provided a study Android smartphone. This information was recorded by the research team in the enrollment session.

#### Smartphone brand

The brand of the participant’s smartphone used in the study was also recorded in the enrollment session.

#### Baseline depression symptom severity

Depressive symptom severity was estimated by the PHQ8 survey administered through the study app at the time of enrollment and every subsequent two weeks. The PHQ8 contains 8 questions and the total score of PHQ8 ranges from 0 to 24 with increasing severity of depressive symptoms^66^. We considered the PHQ8 surveys completed at enrollment to represent the participants’ baseline depression severity.

#### Comorbidity and medication

The participant’s comorbidity information related to 19 types of common comorbidities (listed in Supplementary Table 12) was recorded in the enrolment session. Also, participant use of depression medication was recorded at enrollment. For the present analysis, we used a binary variable to indicate whether the participant had comorbidities and whether they were taking depression medication at the time of enrollment.

#### Survey response and completion time

Survey response time is calculated as the time that elapsed between the notification arrival time in the study app and the time at which participants started responding to the survey. Survey completion time was the total time participants spent completing the survey. Several studies suggested that the response time and the speed of answering questions could reflect the participants’ attitude strength to the survey^25–27^. Therefore, we used these two metrics to reflect participants’ interests and enthusiasm about the study and test whether they are linked to long-term engagement patterns. Both metrics were calculated for the two surveys (PHQ8 and RSES).

### Statistical analysis

We used a survival modeling approach^70^ to assess participants’ overall duration in the study (retention). Survival models are commonly used in medical research for exploring associations between the time passed before some events occur and one or more predictor variables^71^. The survival models were also used in a recent participant retention study^21^. In our participant retention analysis, the event is the participant disengaging from the study app (stopping contributing data to the study) and the elapsed time is the duration in the study (described above).

#### Data harmonization

We conducted our survival analysis on two separate observation periods. The first, referred to as our primary cohort, has an observation period of 43 weeks. This period matches the number of weeks between the date the last patient enrolled in RADAR-MDD (June 2020) and the end of data collection in RADAR-MDD (April 2021). Therefore, it represents the common maximum theoretical survival observation period for all participants enrolled in the RADAR-MDD study. We used this cohort for the presented primary analysis. We also defined a secondary cohort with a survival observation period of 94 weeks. This longer period of observation represents the maximum survival observation period for 50% of participants enrolled in the RADAR-MDD study. Using this secondary cohort, we aimed to investigate even longer-term participant behavior patterns in remote studies.

#### Participant retention analysis

We first used Kaplan-Meier curves^72^ to measure the overall participant retention rates over the two observation periods for three data streams, respectively. To further assess the joint effect of multiple variables of interest on participants’ retention in the study, we used the Cox Proportional-Hazard (CoxPH) model^34^. We considered the baseline PHQ8 score, comorbidity, depression medication, socio-demographics (age, gender, marital status, children, years in education, annual income, and accommodation type), phone status, phone brand, and the study site as predictor variables. If the duration in the study of a participant is equal to the cutoff observation period, we consider the participant to be engaged in the study (no event). To minimize undue influence associated with periodic disengagement (i.e., some participants stop engaging for a while, then re-engage), the right-censoring method^72^ was used for participants whose duration in the study was less than the observation period. We relaxed the determination of the event by considering 4 more weeks after the cut-off day. For example, if a participant’s last active survey was completed on Week 30 within the first 43 weeks (using the primary cutoff observation period), but if they completed more active surveys between Week 44-Week 47 (4-week extension), we still considered this participant was engaged in contributing active data in the study (no event). Otherwise, if there was no completed survey during 4 weeks after the cut-off day, we considered this participant stopped contributing active data in the study (the event happened i.e., participant stopped contributing Phone-Active data to the study). Note, the same methodology was used to counter periodic disengagement in the Phone-Passive, and Fitbit-Passive data. To assess the joint effect of multiple variables of interest on retention, we used separate CoxPH models for Phone-Active, Phone-Passive, and Fitbit-Passive data across the two observation periods (43 weeks and 94 weeks).

The CoxPH model provides an estimate of the hazard ratio (HR) for each predictor. The HR of a predictor greater than 1 indicates the variable is associated with a higher risk of participants not contributing data to the study thus negatively impacting participant retention in the study. The assumption of CoxPH regression^73^, i.e., HR for all predictors should be constant over time, was tested using the scaled Schoenfeld residuals^74^. For predictors that violated the assumption, an interaction term of the covariate with a split time variable was used^35,36^.

### Clustering analysis

We used an unsupervised K-means clustering method^75^ to explore potential latent patterns of participant long-term engagement in the study using the longitudinal data-availability vector (defined above). The elbow method was used to determine the optimal number of clusters^75^. The Kruskal-Wallis test was used to assess any potential enrichment of variables of interest (described above) across the clusters^33^. The same approach was applied to the three data streams and across the two observation periods. Transitions of participants in clusters across the three data streams were recorded and visualized by Sankey diagrams^76^.

### Reporting summary

Further information on research design is available in the Nature Research Reporting Summary linked to this article.

## Supplementary information

Supplementary File

Reporting Summary

## Data Availability

The datasets used for the present study can be made available through reasonable requests to the RADAR-CNS consortium. Please email the corresponding author for details.

## Code availability

The complete code used for the analysis is available through a GitHub code repository (https://github.com/kcl-bhi/RADAR_MDD_Pariticipant_Engagement_and_Retention_Analysis).

## References

[1] Cai N, Choi KW, Fried EI (2020). Reviewing the genetics of heterogeneity in depression: operationalizations, manifestations and etiologies. Hum. Mol. Genet..

[2] Klasen F (2015). Risk and protective factors for the development of depressive symptoms in children and adolescents: results of the longitudinal BELLA study. Eur. Child Adolesc. Psychiat..

[3] Snyder M, Zhou W (2019). Big data and health. Lancet Digit. Health.

[4] Gilchrist G, Gunn J (2007). Observational studies of depression in primary care: what do we know?. BMC Family Pract..

[5] Liew CS, Wah TY, Shuja J, Daghighi B (2015). Mining personal data using smartphones and wearable devices: a survey. Sensors.

[6] Bardram JE, Matic A (2020). A decade of ubiquitous computing research in mental health. IEEE Pervasive Comput..

[7] Bailon C (2019). Smartphone-based platform for affect monitoring through flexibly managed experience sampling methods. Sensors.

[8] Ranjan Y (2019). RADAR-base: open source mobile health platform for collecting, monitoring, and analyzing data using sensors, wearables, and mobile devices. JMIR mHealth uHealth.

[9] Pratap A (2018). Using mobile apps to assess and treat depression in Hispanic and Latino populations: fully remote randomized clinical trial. J. Med. Internet. Res..

[10] Matcham F (2019). Remote assessment of disease and relapse in major depressive disorder (RADAR-MDD): a multi-centre prospective cohort study protocol. BMC Psychiat..

[11] Luik AI (2015). 24-h activity rhythm and sleep disturbances in depression and anxiety: a population‐based study of middle‐aged and older persons. Depress. Anxiety.

[12] Cho YM (2016). A cross-sectional study of the association between mobile phone use and symptoms of ill health. Environ. Health Toxicol..

[13] Zhang Y (2021). Relationship between major depression symptom severity and sleep collected using a wristband wearable device: multicenter longitudinal observational study. JMIR mHealth uHealth.

[14] Zhang Y (2021). Predicting depressive symptom severity through individuals’ nearby bluetooth device count data collected by mobile phones: preliminary longitudinal study. JMIR Mhealth Uhealth.

[15] Laiou P (2022). The association between home stay and symptom severity in major depressive disorder: preliminary findings from a multicenter observational study using geolocation data from smartphones. JMIR Mhealth Uhealth.

[16] Zhang Y (2022). Longitudinal relationships between depressive symptom severity and phone-measured mobility: dynamic structural equation modeling study. JMIR Ment Health.

[17] Zhang Y (2022). Associations between depression symptom severity and daily-life gait characteristics derived from long-term acceleration signals in real-world settings: retrospective analysis. JMIR Mhealth Uhealth.

[18] Moore S, Tassé A-M, Thorogood A, Winship I, Doerr M (2017). Consent processes for mobile app mediated research: systematic review. JMIR mHealth uHealth.

[19] Druce KL, Dixon WG, McBeth J (2019). Maximizing engagement in mobile health studies: lessons learned and future directions. Rheum. Dis. Clin. North Am..

[20] De Angel V (2022). Digital health tools for the passive monitoring of depression: a systematic review of methods. NPJ Digit. Med..

[21] Pratap A (2020). Indicators of retention in remote digital health studies: a cross-study evaluation of 100,000 participants. NPJ Digit. Med..

[22] Simblett S (2018). Barriers to and facilitators of engagement with remote measurement technology for managing health: systematic review and content analysis of findings. J. Med. Internet Res..

[23] O’connor S (2016). Understanding factors affecting patient and public engagement and recruitment to digital health interventions: a systematic review of qualitative studies. BMC Med. Inform. Decis. Mak..

[24] Quisel T, Foschini L, Zbikowski SM, Juusola JL (2019). The association between medication adherence for chronic conditions and digital health activity tracking: retrospective analysis. J. Med. Internet Res..

[25] Bassili JN (1996). Meta-judgmental versus operative indexes of psychological attributes: The case of measures of attitude strength. J. Personal. Social Psychol..

[26] Heerwegh D (2003). Explaining response latencies and changing answers using client-side paradata from a web survey. Social Sci. Comput. Rev..

[27] Fazio RH, Powell MC, Herr PM (1983). Toward a process model of the attitude–behavior relation: accessing one’s attitude upon mere observation of the attitude object. J. Personal. Social Psychol..

[28] Matcham, F. et al. Remote Assessment of Disease and Relapse in Major Depressive Disorder (RADAR-MDD): recruitment, retention, and data availability in a longitudinal remote measurement study. *BMC Psychiat.* **22**, 1–19 (2022). 10.1186/s12888-022-03753-1 PMC8860359 35189842

[29] Albert PR (2015). Why is depression more prevalent in women?. J. Psychiat. Neurosci. JPN.

[30] Noble RE (2005). Depression in women. Metabolism.

[31] Salk RH, Hyde JS, Abramson LY (2017). Gender differences in depression in representative national samples: meta-analyses of diagnoses and symptoms. Psychol. Bullet..

[32] Van de Velde S, Bracke P, Levecque K (2010). Gender differences in depression in 23 European countries. Cross-national variation in the gender gap in depression. Social Sci. Med..

[33] Ostertagova, E., Ostertag, O. & Kováč, J. in *Applied Mechanics and Materials*. 115–120 (Trans Tech Publ).

[34] Kumar D, Klefsjö B (1994). Proportional hazards model: a review. Reliab. Eng. Syst. Safety.

[35] Ata N, Sözer MT (2007). Cox regression models with nonproportional hazards applied to lung cancer survival data. Hacettepe J. Math. Stat..

[36] Borucka, J. Extensions of Cox model for non-proportional hazards purpose. *Ekonometria*, 85–101 (2014).

[37] Wu, J. in *Advances in K-means Clustering* 1–16 (Springer, 2012).

[38] Chien I (2020). A machine learning approach to understanding patterns of engagement with internet-delivered mental health interventions. JAMA Network Open.

[39] Tariq S, Woodman J (2013). Using mixed methods in health research. JRSM Short Rep..

[40] Papoutsi C, Wherton J, Shaw S, Morrison C, Greenhalgh T (2021). Putting the social back into sociotechnical: case studies of co-design in digital health. J. Am. Med. Inform. Assoc..

[41] Shaw J (2018). Beyond “implementation”: digital health innovation and service design. NPJ Digit. Med..

[42] Awais M (2020). Healthcare professional in the loop (HPIL): classification of standard and oral cancer-causing anomalous regions of oral cavity using textural analysis technique in autofluorescence imaging. Sensors.

[43] Goodday SM (2021). An alternative to the light touch digital health remote study: the stress and recovery in frontline COVID-19 Health Care Workers Study. JMIR Form. Res..

[44] Pratap A (2019). The accuracy of passive phone sensors in predicting daily mood. Depress. Anxiety.

[45] Dineley, J. et al. in 22nd Annual Conference of the International Speech Communication Association, INTERSPEECH 2021. 631–635 (International Speech Communication Association).

[46] Li, S. X. et al. Recruitment and Retention in Remote Research: Learnings From a Large, Decentralized Real-world Study. JMIR Form Res **6**, e40765 (2022). 10.2196/40765 PMC9706389 36374539

[47] Althubaiti A (2016). Information bias in health research: definition, pitfalls, and adjustment methods. J. Multidiscipl. Healthcare.

[48] Bentley JP, Thacker PG (2004). The influence of risk and monetary payment on the research participation decision making process. J. Med. Ethics.

[49] Birnbaum F, Lewis DM, Rosen R, Ranney ML (2015). Patient engagement and the design of digital health. Acad. Emerg. Med. Official J. Soc. Acad. Emerg. Med..

[50] Kaye J (2012). From patients to partners: participant-centric initiatives in biomedical research. Nat. Rev. Genet..

[51] Anderson N, Bragg C, Hartzler A, Edwards K (2012). Participant-centric initiatives: tools to facilitate engagement in research. Appli. Transl. Genom..

[52] Forsat ND, Palmowski A, Palmowski Y, Boers M, Buttgereit F (2020). Recruitment and retention of older people in clinical research: a systematic literature review. J. Am. Geriatrics Soc..

[53] Mody L (2008). Recruitment and retention of older adults in aging research: (see editorial comments by Dr. Stephanie Studenski, pp 2351–2352). J. Am. Geriatri. Soc..

[54] Pywell J, Vijaykumar S, Dodd A, Coventry L (2020). Barriers to older adults’ uptake of mobile-based mental health interventions. Digit. Health.

[55] Arean PA (2016). The use and effectiveness of mobile apps for depression: results from a fully remote clinical trial. J. Med. Internet Res..

[56] Difrancesco S (2019). Sleep, circadian rhythm, and physical activity patterns in depressive and anxiety disorders: a 2‐week ambulatory assessment study. Depressi. Anxiety.

[57] Lu J (2018). Joint modeling of heterogeneous sensing data for depression assessment via multi-task learning. Proc. ACM Interacti. Mobile Wearable Ubiquitous Technol..

[58] Kroenke K, Spitzer RL, Williams JB (2001). The PHQ‐9: validity of a brief depression severity measure. J. Gen. Intern. Med..

[59] Beard C, Hsu K, Rifkin L, Busch A, Björgvinsson T (2016). Validation of the PHQ-9 in a psychiatric sample. J. Affect. Disord..

[60] Cameron IM (2011). Measuring depression severity in general practice: discriminatory performance of the PHQ-9, HADS-D, and BDI-II. Br. J. Gen. Pract..

[61] Rohani DA, Faurholt-Jepsen M, Kessing LV, Bardram JE (2018). Correlations between objective behavioral features collected from mobile and wearable devices and depressive mood symptoms in patients with affective disorders: systematic review. JMIR mHealth uHealth.

[62] Wang, R. et al. in Proceedings of the 2014 ACM international joint conference on pervasive and ubiquitous computing. 3–14.

[63] Inoue T (2012). Utility and limitations of PHQ-9 in a clinic specializing in psychiatric care. BMC Psychiatry.

[64] Regier DA (2013). DSM-5 field trials in the United States and Canada, Part II: test-retest reliability of selected categorical diagnoses. Am. J Psychiatry.

[65] Simblett S (2019). Barriers to and facilitators of engagement with mHealth technology for remote measurement and management of depression: qualitative analysis. JMIR Mhealth Uhealth.

[66] Kroenke K (2009). The PHQ-8 as a measure of current depression in the general population. J. Affect. Disord..

[67] Greenberger E, Chen C, Dmitrieva J, Farruggia SP (2003). Item-wording and the dimensionality of the Rosenberg self-esteem scale: do they matter?. Personal. Individ. Differ..

[68] Baumel A, Muench F, Edan S, Kane JM (2019). Objective user engagement with mental health apps: systematic search and panel-based usage analysis. J. Med. Internet Res..

[69] Torous J, Lipschitz J, Ng M, Firth J (2020). Dropout rates in clinical trials of smartphone apps for depressive symptoms: a systematic review and meta-analysis. J. Affect. Disord..

[70] Bewick V, Cheek L, Ball J (2004). Statistics review 12: survival analysis. Critical Care.

[71] Singer JD, Willett JB (1991). Modeling the days of our lives: using survival analysis when designing and analyzing longitudinal studies of duration and the timing of events. Psychol. Bullet..

[72] Rich JT (2010). A practical guide to understanding Kaplan-Meier curves. Otolaryngology—Head and Neck Surgery.

[73] Kleinbaum, D. G. & Klein, M. in *Survival analysis* 161–200 (Springer, 2012).

[74] Grambsch PM, Therneau TM (1994). Proportional hazards tests and diagnostics based on weighted residuals. Biometrika.

[75] Syakur, M., Khotimah, B., Rochman, E. & Satoto, B. D. in *IOP Conference Series: Materials Science and Engineering*. 012017 (IOP Publishing).

[76] Schmidt M (2008). The Sankey diagram in energy and material flow management: part II: methodology and current applications. J. Indus. Ecol..

