# Supplementary material for: Long-term participant retention and engagement patterns in an app and wearable-based multinational remote digital depression study
Source: NPJ Digit Med. 2023 Feb 17;6:25. doi: 10.1038/s41746-023-00749-3 (PMC9938183; doi:10.1038/s41746-023-00749-3)
Supplement: Supplementary file 1 — Supplementary File [file 41746_2023_749_MOESM1_ESM.pdf]

Supplementary Table 1. A summary of characteristics of 313 participants with a longer observation period (94 weeks) in the RADAR-MDD study, with comparisons across the three study sites using the Kruskal-Wallis tests. Note, ethnicity data was not collected for participants recruited at the CIBER site.

**Characteristics**	**Total**	**KCL**	**CIBER**	**VUMC**	**p value**
**Number of participants, n**	313	206	91	16	
**Age (median [IQR])**	51.00 [37.00, 59.00]	47.00 [32.00, 58.00]	54.00 [47.50, 60.00]	39.50 [33.50, 52.25]	<.001
**Female, n (%)**	235 (75.1)	155 (75.2)	67 (73.6)	13 (81.3)	.81
**Marital status, n (%)**					.06
Single/separated/divorced/widowed	155 (49.5)	109 (52.9)	36 (39.6)	10 (62.5)	
Married/cohabiting/LTR	158 (50.5)	97 (47.1)	55 (60.4)	6 (37.5)	
**Ethnicity, n (%)**					<.001
White	188 (84.7)	174 (84.5)	-	14 (87.5)	
Black	9 (4.1)	8 (3.9)	-	1 (6.2)	
Asian	9 (4.1)	9 (4.4)	-	0 (0)	
Other	16 (7.2)	15 (7.3)	-	1 (6.2)	
**Employed, n (%)**	135 (43.1)	106 (51.5)	19 (20.9)	10 (62.5)	<.001
**Having children, n (%)**	173 (55.3)	97 (47.1)	70 (76.9)	6 (37.5)	<.001
**Years in education (median [IQR])**	15.00 [12.00, 18.00]	17.00 [14.00, 19.00]	11.00 [9.00, 14.00]	17.00 [15.00, 21.50]	<.001
**Annual income, n (%)**					.003
<15,000 (£/€)	86 (27.5)	53 (25.7)	32 (35.2)	1 (6.3)	
15,000-55,000 (£/€)	181 (57.8)	116 (56.3)	53 (58.2)	12 (75.0)	
>55000 (£/€)	44 (14.1)	36 (17.5)	6 (6.6)	2 (12.5)	
**Accommodation, n (%)**					.04
Own outright/with mortgage	177 (56.6)	105 (51.0)	63 (69.2)	9 (56.3)	
Renting	110 (35.1)	82 (39.8)	21 (23.1)	7 (43.8)	
Living rent-free	23 (7.4)	18 (8.7)	5 (5.5)	0 (0)	
**Baseline PHQ8 score (median [IQR])**	11.00 [7.00, 16.00]	8.00 [5.25, 13.00]	15.00 [10.00, 18.00]	9.00 [7.00, 10.00]	<.001
**Having comorbidities, n (%)**	176 (56.2)	113 (54.9)	60 (65.9)	3 (18.8)	.002
**Taking depression medication, n (%)**	208 (66.5)	116 (56.3)	84 (92.3)	8 (50.0)	<.001
**Number of contact logs (median [IQR])**	9.00 [6.00, 14.00]	12.00 [8.00, 16.00]	5.00 [3.00, 8.00]	3.50 [2.75, 5.25]	<.001
**Provided phone, n (%)**	80 (25.6)	71 (34.5)	6 (6.7)	3 (18.8)	<.001
**Smartphone brand, n (%)**					<.001
Motorola	136 (43.7)	109 (53.2)	22 (24.4)	5 (31.2)	
Samsung	85 (27.3)	54 (26.3)	23 (25.6)	8 (50.0)	
Other	90 (28.9)	42 (20.5)	45 (50.0)	3 (18.8)	

Supplementary Table 2. Proportional hazards assumption tests (using the scaled Schoenfeld residuals) for 3 Cox Proportional-Hazards models of Phone-Active, Phone-Passive, and Fitbit-Passive data streams during the first 43 weeks of the RADAR-MDD study, respectively. Except "gender" variable of the Phone-Passive model, all variables passed the assumption. For the Phone-Passive model, an interaction term of the "gender" variable with a split time variable (cut points are 130 and 240) was used.

**Predictor**	**Phone-Active**		**Phone-Passive**		**Fitbit-Passive**	
	χ^2	**p value**	χ^2	**p value**	χ^2	**p value**
Age	0.41	.98	6.14	.19	6.22	.18
Gender	0.63	.43	—	—	0.67	.41
Gender strata by time	—	—	2.91	.41	—	—
Marital status	1.91	.17	2.30	.13	0.77	.38
Employment	1.88	.17	1.96	.16	1.78	.18
Having children	0.12	.73	0.03	.85	1.35	.25
Years in education	2.77	.10	0.05	.82	0.82	.36
Annual income	1.45	.48	1.36	.51	0.66	.72
Accommodation	2.09	.35	1.95	.38	2.32	.31
Baseline PHQ8 score	0.24	.63	0.77	.38	0.38	.54
Having comorbidities	1.87	.17	0.002	.97	0.04	.84
Taking depression medication	0.68	.41	0.87	.35	0.10	.75
Study Site	0.54	.76	4.07	.13	4.59	.10
Brand of smartphone	0.39	.82	4.13	.13	0.57	.75
Phone status	0.06	.80	0.28	.60	0.61	.44
GLOBAL	13.52	.89	27.75	.23	22.05	.40

Supplementary Table 3. The estimates and 95% confidence intervals of hazard ratio (HR) of variables in the Cox models for assessing the impact of multiple variables of interest on the participant retention time in the first 94 weeks of the study. In the Phone-Active model, the "age" variable was split by time (the cut point is 320). For 320-660 days, compared with <30, HR of 30-39 = 0.2 (0.07-0.56) (p < .001), HR of 40-49 = 0.56 (0.28-1.15) (p = .11), HR of 50-59 = 0.24 (0.1-0.53) (p < .001), HR of >60 = 0.17 (0.07-0.41) (p <.001). For 1-319 days, all HRs of "age" were not statistically significant. Similarly, in the Fitbit-Passive model, the "children" variable was also split by time (the cut is 450); HRs of "children" were not significant in both periods.

Variables		**Phone-Active**		**Phone-Passive**		**Fitbit-Passive**	
		**HR (95% CI)**	**p**	**HR (95% CI)**	**p**	**HR (95% CI)**	**p**
Age	<30	—	—	Reference	—	Reference	—
	30-39	—	—	0.79 (0.43-1.43)	.43	0.64 (0.33-1.25)	.19
	40-49	—	—	0.65 (0.35-1.22)	.18	0.48 (0.24-0.96)	.04
	50-59	—	—	0.71 (0.37-1.36)	.30	0.4 (0.2-0.83)	.01
	>60	—	—	0.38 (0.19-0.77)	.01	0.38 (0.17-0.83)	.02
Gender	Female	Reference	—	Reference	—	Reference	—
	Male	0.74 (0.47-1.16)	.19	0.7 (0.47-1.05)	.09	0.86 (0.55-1.35)	.52
Marital status	Single	Reference	—	Reference	—	Reference	—
	Married	1.06 (0.68-1.66)	.79	1.06 (0.72-1.57)	.76	0.88 (0.56-1.39)	.58
Employment	No	Reference	—	Reference	—	Reference	—
	Yes	0.66 (0.42-1.04)	.08	0.78 (0.52-1.17)	.24	0.68 (0.43-1.08)	.10
Having children	No	Reference	—	Reference	—	—	—
	Yes	1.08 (0.67-1.75)	.75	1.05 (0.68-1.62)	.81	—	—
Years in education		0.99 (0.95-1.03)	.69	1.03 (1-1.06)	.06	1.01 (0.97-1.04)	.68
Annual income (£/€)	<15,000	Reference	—	Reference	—	Reference	—
	15,000-55,000	1.45 (0.91-2.31)	.12	1.36 (0.89-2.09)	.16	1.06 (0.65-1.7)	.82
	>55000	0.98 (0.46-2.11)	.96	0.9 (0.46-1.74)	.75	0.61 (0.27-1.39)	.24
Accommodation	Own outright	Reference	—	Reference	—	Reference	—
	Renting	1.35 (0.88-2.06)	.17	1.34 (0.91-1.95)	.13	1.15 (0.74-1.78)	.53
	Living rent-free	0.88 (0.4-1.91)	.74	0.85 (0.4-1.82)	.68	0.65 (0.28-1.49)	.30
PHQ8 score		1.01 (0.97-1.05)	.58	1.01 (0.98-1.04)	.63	1 (0.96-1.04)	.88
Having comorbidities	No	Reference	—	Reference	—	Reference	—
	Yes	0.8 (0.53-1.2)	.27	0.75 (0.52-1.08)	.12	0.79 (0.52-1.19)	.26
Medication	No	Reference	—	Reference	—	Reference	—
	Yes	0.79 (0.52-1.21)	.28	1.29 (0.86-1.93)	.22	0.98 (0.63-1.52)	.92
Study Site	CIBER	Reference	—	Reference	—	Reference	—
	KCL	0.66 (0.4-1.08)	.10	0.77 (0.49-1.21)	.26	0.71 (0.42-1.18)	.19
	VUMC	0.66 (0.27-1.61)	.37	0.91 (0.42-1.99)	.82	0.58 (0.22-1.57)	.29
Brand of smartphone	Other	Reference	—	Reference	—	Reference	—
	Motorola	0.6 (0.34-1.05)	.07	0.47 (0.29-0.76)	<.001	1 (0.58-1.71)	.99
	Samsung	0.85 (0.53-1.37)	.51	0.71 (0.47-1.07)	.10	0.84 (0.51-1.38)	.50
Phone status	Own phone	Reference	—	Reference	—	Reference	—
	Provided phone	1.4 (0.92-2.12)	.12	0.88 (0.58-1.33)	.54	0.91 (0.59-1.4)	.65

Supplementary Table 4. Proportional hazards assumption tests (using the scaled Schoenfeld residuals) for 3 Cox Proportional-Hazards models of Phone-Active, Phone-Passive, and Fitbit-Passive data streams during the first 94 weeks of the RADAR-MDD study, respectively. All 3 Cox models passed the global proportional hazards assumption tests. Except "age" variable of the Phone-Active model and "having children" variable of Fitbit-Passive" model, all variables passed the assumption. For the Phone-Active model, an interaction term of the "age" variable with a split time variable (the cut point is 320) was used. For the Fitbit-Passive model, an interaction term of the "having children" variable with a split time variable (the cut point is 450) was used.

**Predictor**	**Phone-Active**		**Phone-Passive**		**Fitbit-Passive**	
	χ^2	**p value**	χ^2	**p value**	χ^2	**p value**
Age	—	—	1.93	.75	4.61	.33
Age strata by time	2.19	.98	—	—	—	—
Gender	1.79	.18	2.22	.14	2.13	.14
Marital status	0.23	.63	0.01	.91	0.64	.42
Employment	1.06	.30	0.01	.92	3.28	.07
Having children	0.58	.45	1.23	.27	—	—
Having children strata by time	—	—	—	—	1.44	.49
Years in education	0.02	.89	0.74	.39	0.49	.48
Annual income	0.51	.78	0.33	.85	1.90	.39
Accommodation	1.91	.38	1.48	.48	1.05	.59
Baseline PHQ8 score	0.28	.60	1.36	.24	0.20	.66
Having comorbidities	1.83	.18	0.09	.76	0.89	.34
Taking depression medication	2.97	.09	0.64	.42	0.01	.94
Study Site	1.77	.41	0.87	.65	0.84	.66
Brand of smartphone	0.71	.70	0.01	.99	1.83	.40
Phone status	0.22	.64	0.10	.75	1.97	.16
GLOBAL	17.68	.86	13.87	.87	22.75	.42

Supplementary Table 5. A summary of participants' characteristics across three distinct engagement subgroups of Phone-Active data for the first 43 weeks of the RADAR-MDD study. The median and interquartile range (IQR) of continuous variables and the count number and percentage of category variables are reported in the table. All p values were assessed using the Kruskal-Wallis tests.

**Characteristics**	**C1**	**C2**	**C3**	**p value**
**Number of participants, n**	231	179	204	
**Age**	53.00 [34.00, 61.50]	45.00 [31.00, 55.50]	48.00 [32.00, 57.25]	.003
**Male, n (%)**	52 (22.5)	42 (23.5)	55 (27.0)	.53
**Number of biweekly surveys**	20.00 [18.00, 21.00]	13.00 [11.00, 15.00]	4.00 [1.00, 6.00]	<.001
**PHQ8 response time (minutes)**	73.68 [31.31, 215.77]	148.08 [54.05, 322.27]	302.36 [122.30, 527.10]	<.001
**PHQ8 completion time (seconds)**	50.29 [37.92, 68.96]	49.42 [40.01, 66.95]	61.56 [46.12, 83.00]	<.001
**RSES response time (minutes)**	67.94 [24.11, 198.42]	134.96 [44.55, 304.56]	274.09 [108.18, 518.50]	<.001
**RSES completion time (seconds)**	54.54 [43.78, 72.39]	55.75 [43.99, 74.28]	69.24 [50.34, 100.52]	<.001
**Site, n (%)**				<.001
CIBER	47(20.3)	48 (26.8)	51 (25.0)	
KCL	119 (51.5)	102 (57.0)	129 (63.2)	
VUMC	65 (28.1)	29 (16.2)	24 (11.8)	
**Married Status, n (%)**				.24
Single/separated/divorced/widowed	114 (49.4)	97 (54.2)	117 (57.4)	
Married/cohabiting/LTR	117 (50.6)	82 (45.8)	87 (42.6)	
**Years in education**	16.00 [12.00, 19.00]	16.00 [13.00, 19.00]	15.00 [12.00, 19.00]	.90
**Having children, n (%)**	116 (50.2)	88 (49.2)	100 (49.0)	.78
**Employed, n (%)**	90 (39.0)	80 (44.7)	88 (43.1)	.06
**Annual income, n (%)**				.73
<15,000 (£/€)	48 (20.8)	48 (26.8)	56 (27.5)	
15,000-55,000 (£/€)	135 (58.4)	102 (57.0)	111 (54.4)	
more than 55000 (£/€)	40 (17.3)	25 (14.0)	33 (16.2)	
**Accommodation, n (%)**				.47
Own outright/with mortgage	131 (56.7)	90 (50.3)	102 (50.0)	
Renting	82 (35.5)	73 (40.8)	81 (39.7)	
Living rent-free	13 (5.6)	15 (8.4)	18 (8.8)	
**Baseline PHQ8 score**	9.00 [6.00, 15.00]	10.00 [8.00, 15.00]	13.00 [7.00, 17.00]	.003
**Having comorbidities, n (%)**	109 (47.2)	86 (48.0)	116 (56.9)	.09
**Taking depression medication, n (%)**	145 (62.8)	126 (70.4)	129 (63.2)	.22
**Number of contact logs**	3.00 [2.00, 5.00]	5.00 [3.00, 7.00]	5.00 [2.00, 9.00]	<.001
**Provided phone, n (%)**	55 (24.3)	40 (22.5)	56 (28.3)	.41
**Brand of smartphone, n (%)**				.56
Motorola	99 (43.4)	66 (37.1)	75 (37.7)	
Samsung	72 (31.6)	56 (31.5)	66 (33.2)	
Other	57 (25.0)	56 (31.5)	58 (29.1)	

Supplementary Table 6. A summary of participants' characteristics across three distinct engagement subgroups of Phone-Passive data for the first 43 weeks of the RADAR-MDD study. The median and interquartile range (IQR) of continuous variables and the count number and percentage of category variables are reported in the table. All p values were assessed using the Kruskal-Wallis tests.

**Characteristics**	**C1**	**C2**	**C3**	**p value**
**Number of participants, n**	259	148	207	
**Age**	52.00 [36.50, 61.00]	46.50 [30.75, 56.25]	46.00 [30.50, 57.50]	.01
**Male, n (%)**	66 (25.5)	36 (24.3)	47 (22.7)	.79
**Days with phone passive data**	283.00 [257.00, 298.00]	167.00 [142.25, 205.25]	32.00 [4.00, 67.50]	<.001
**PHQ8 response time (minutes)**	121.70 [45.13, 327.68]	99.76 [40.89, 289.39]	150.94 [52.23, 340.70]	.21
**PHQ8 completion time (seconds)**	53.63 [41.84, 72.91]	48.08 [36.46, 66.11]	55.29 [39.75, 74.47]	.01
**RSES response time (minutes)**	116.13 [36.36, 347.98]	81.50 [31.10, 254.39]	160.42 [39.65, 321.28]	.14
**RSES completion time (seconds)**	61.08 [47.33, 83.46]	51.87 [41.31, 70.32]	63.55 [46.68, 82.90]	.001
**Site, n (%)**				<.001
CIBER	60 (23.2)	24 (16.2)	62 (30.0)	
KCL	165 (63.7)	82 (55.4)	103 (49.8)	
VUMC	34 (13.1)	42 (28.4)	42 (20.3)	
**Married Status, n (%)**				.26
Single/separated/divorced/widowed	131 (50.6)	77 (52.0)	120 (58.0)	
Married/cohabiting/LTR	128 (49.4)	71 (48.0)	87 (42.0)	
**Years in education**	15.00 [12.00, 18.00]	17.00 [14.00, 20.00]	16.00 [12.00, 19.00]	.005
**Having children, n (%)**	142 (54.8)	66 (44.6)	96 (46.4)	.15
**Employed, n (%)**	106 (40.9)	64 (43.2)	88 (42.5)	.87
**Annual income, n (%)**				.80
<15,000 (£/€)	65 (25.1)	31 (20.9)	56 (27.1)	
15,000-55,000 (£/€)	151 (58.3)	83 (56.1)	114 (55.1)	
more than 55000 (£/€)	37 (14.3)	29 (19.6)	32 (15.5)	
**Accommodation, n (%)**				.08
Own outright/with mortgage	150 (57.9)	71 (48.0)	102 (49.3)	
Renting	90 (34.7)	67 (45.3)	79 (38.2)	
Living rent-free	17 (6.6)	7 (4.7)	22 (10.6)	
**Baseline PHQ8 score**	9.00 [6.00, 15.00]	10.00 [7.00, 14.00]	12.00 [8.00, 17.00]	.001
**Having comorbidities, n (%)**	135 (52.1)	75 (50.7)	101 (48.8)	.78
**Taking depression medication, n (%)**	161 (62.2)	95 (64.2)	144 (69.6)	.24
**Number of contact logs**	5.00 [2.00, 7.00]	4.00 [2.00, 7.00]	4.00 [2.00, 7.00]	.30
**Provided phone, n (%)**	84 (32.6)	44 (29.9)	23 (11.7)	< .001
**Brand of smartphone, n (%)**				<.001
Motorola	147 (57.0)	63 (42.9)	30 (15.0)	
Samsung	71 (27.5)	55 (37.4)	68 (34.0)	
Other	40 (15.5)	29 (19.7)	102 (51.0)	

Supplementary Table 7. A summary of participants' characteristics across three distinct engagement subgroups of Fitbit-Passive data for the first 43 weeks of the RADAR-MDD study. The median and interquartile range (IQR) of continuous variables and the count number and percentage of category variables are reported in the table. All p values were assessed using the Kruskal-Wallis tests.

Characteristics	C1	C2	C3	p value
**Number of participants, n**	407	99	108	
**Age**	48.00 [32.00, 58.50]	45.00 [31.50, 54.00]	51.50 [36.00, 61.00]	.06
**Male, n (%)**	99 (24.3)	26 (26.3)	24 (22.2)	.79
**Days with Fitbit passive data**	294.00 [274.00, 301.00]	156.00 [132.00, 190.00]	18.00 [0.00, 67.00]	<.001
**PHQ8 response time (minutes)**	113.51 [38.68, 288.84]	170.78 [53.70, 470.75]	161.81 [65.91, 410.24]	.007
**PHQ8 completion time (seconds)**	50.28 [39.19, 66.99]	52.80 [37.71, 81.08]	64.20 [50.71, 84.31]	<.001
**RSES response time (minutes)**	97.68 [29.95, 288.16]	143.47 [56.43, 423.82]	177.56 [65.53, 381.02]	.009
**RSES completion time (seconds)**	56.40 [44.29, 73.28]	60.74 [44.95, 87.61]	69.43 [53.61, 96.26]	<.001
**Site, n (%)**				<.001
CIBER	77 (18.9)	29 (29.3)	40 (37.0)	
KCL	237 (58.2)	54 (54.5)	59 (54.6)	
VUMC	93 (22.9)	16 (16.2)	9 (8.3)	
**Married Status, n (%)**				.06
Single/separated/divorced/widowed	204 (50.1)	61 (61.6)	63 (58.3)	
Married/cohabiting/LTR	203 (49.9)	38 (38.4)	45 (41.7)	
**Years in education**	16.00 [13.00, 19.00]	15.00 [13.00, 20.00]	14.50 [11.00, 18.00]	.004
**Having children, n (%)**	191 (46.9)	48 (48.5)	65 (60.2)	.03
**Employed, n (%)**	179 (44.0)	37 (37.4)	42 (38.9)	.47
**Annual income, n (%)**				.08
<15,000 (£/€)	90 (22.1)	35 (35.4)	27 (25.0)	
15,000-55,000 (£/€)	232 (57.0)	53 (53.5)	63 (58.3)	
more than 55000 (£/€)	75 (18.4)	8 (8.1)	15 (13.9)	
**Accommodation, n (%)**				.30
Own outright/with mortgage	215 (52.8)	44 (44.4)	64 (59.3)	
Renting	157 (38.6)	46 (46.5)	33 (30.6)	
Living rent-free	30 (7.4)	8 (8.1)	8 (7.4)	
**Baseline PHQ8 score**	9.00 [6.00, 15.00]	11.00 [7.00, 16.00]	13.00 [9.00, 17.50]	<.001
**Having comorbidities, n (%)**	197 (48.4)	49 (49.5)	65 (60.2)	.09
**Taking depression medication, n (%)**	264 (64.9)	63 (63.6)	73 (67.6)	.82
**Number of contact logs**	4.00 [2.00, 7.00]	5.00 [3.00, 8.00]	4.00 [2.00, 6.50]	.09
**Provided phone, n (%)**	102 (25.3)	27 (27.6)	22 (21.8)	.63
**Brand of smartphone, n (%)**				.047
Motorola	168 (41.6)	39 (39.4)	33 (32.4)	
Samsung	137 (33.9)	27 (27.3)	30 (29.4)	
Other	99 (24.5)	33 (33.3)	39 (38.2)	

Supplementary Table 8. A summary of participants' characteristics across four distinct engagement subgroups of Phone-Active data for the first 94 weeks of the RADAR-MDD study. The median and interquartile range (IQR) of continuous variables and the count number and percentage of category variables are reported in the table. All p values were assessed using the Kruskal-Wallis tests.

**Characteristics**	**C1**	**C2**	**C3**	**C4**	**p**
**Number of participants, n**	82	63	59	109	
**Age**	56.00 [46.25, 63.00]	49.00 [32.50, 59.00]	48.00 [30.00, 57.50]	47.00 [35.00, 54.00]	<.001
**Male, n (%)**	24 (29.3)	15 (23.8)	13 (22.0)	26 (23.9)	.75
**Number of biweekly surveys**	41.00 [37.25, 44.00]	25.00 [22.00, 30.00]	18.00 [14.50, 24.00]	5.00 [1.00, 8.00]	<.001
**PHQ8 response time (minutes)**	84.20 [31.44, 204.10]	114.91 [71.77, 275.07]	158.96 [48.80, 338.91]	185.82 [71.58, 469.91]	.01
**PHQ8 completion time (seconds)**	50.78 [37.80, 66.44]	47.26 [38.60, 57.76]	49.67 [39.45, 64.32]	55.58 [41.74, 81.56]	.04
**RSES response time (minutes)**	62.88 [25.62, 195.36]	104.12 [41.94, 359.53]	176.02 [40.42, 351.18]	194.83 [53.30, 457.22]	.008
**RSES completion time (seconds)**	52.23 [42.13, 71.35]	52.09 [44.01, 67.51]	54.67 [42.18, 69.99]	66.35 [49.14, 98.78]	<.001
**Site, n (%)**					.001
CIBER	27 (32.9)	14 (22.2)	19 (32.2)	31 (28.4)	
KCL	45 (54.9)	49 (77.8)	35 (59.3)	77 (70.6)	
VUMC	10 (12.2)	0 (0.0)	5 (8.5)	1 (0.9)	
**Married Status, n (%)**					.43
Single/separated/divorced/widowed	35 (42.7)	33 (52.4)	28 (47.5)	59 (54.1)	
Married/cohabiting/LTR	47 (57.3)	30 (47.6)	31 (52.5)	50 (45.9)	
**Years in education**	15.50 [12.00, 18.00]	16.00 [12.00, 19.00]	15.00 [12.00, 18.00]	15.00 [12.00, 19.00]	.87
**Having children, n (%)**	32 (39.0)	25 (39.7)	28 (47.5)	54 (49.5)	.47
**Employed, n (%)**	28 (34.1)	34 (54.0)	25 (42.4)	48 (44.0)	.13
**Annual income, n (%)**					.84
<15,000 (£/€)	20 (24.4)	18 (28.6)	18 (30.5)	30 (27.5)	
15,000-55,000 (£/€)	50 (61.0)	38 (60.3)	34 (57.6)	59 (54.1)	
more than 55000 (£/€)	12 (14.6)	7 (11.1)	6 (10.2)	19 (17.4)	
**Accommodation, n (%)**					.08
Own outright/with mortgage	58 (70.7)	33 (52.4)	31 (52.5)	55 (50.5)	
Renting	20 (24.4)	28 (44.4)	22 (37.3)	40 (36.7)	
Living rent-free	3 (3.7)	2 (3.2)	5 (8.5)	13 (11.9)	
**Baseline PHQ8 score**	9.00 [5.75, 13.00]	10.00 [7.00, 14.00]	13.00 [8.50, 17.50]	13.00 [7.00, 17.00]	.005
**Having comorbidities, n (%)**	39 (47.6)	39 (61.9)	32 (54.2)	66 (60.6)	.24
**Taking depression medication, n (%)**	57 (69.5)	38 (60.3)	41 (69.5)	72 (66.1)	.65
**Number of contact logs**	7.00 [5.00, 10.00]	11.00 [7.00, 17.00]	9.00 [5.00, 13.50]	10.00 [5.00, 14.00]	.002
**Provided phone, n (%)**	17 (20.7)	17 (27.0)	12 (20.3)	34 (31.5)	.27
**Brand of smartphone, n (%)**					.13
Motorola	45 (55.6)	23 (36.5)	22 (37.3)	46 (42.6)	
Samsung	17 (21.0)	18 (28.6)	15 (25.4)	35 (32.4)	
Other	19 (23.5)	22 (34.9)	22 (37.3)	27 (25.0)	

Supplementary Table 9. A summary of participants' characteristics across four distinct engagement subgroups of Phone-Passive data for the first 94 weeks of the RADAR-MDD study. The median and interquartile range (IQR) of continuous variables and the count number and percentage of category variables are reported in the table. All p values were assessed using the Kruskal-Wallis tests.

Characteristics	C1	C2	C3	C4	*p*
**Number of participants, n**	122	53	61	77	
**Age**	54.00 [41.00, 62.75]	46.00 [35.00, 53.00]	48.00 [37.00, 55.00]	50.00 [36.00, 59.00]	.009
**Male, n (%)**	33 (27.0)	9 (17.0)	16 (26.2)	20 (26.0)	.54
**Days with phone passive data**	607.00 [538.25, 639.00]	417.00 [362.00, 465.00]	230.00 [179.00, 286.00]	31.00 [3.00, 86.00]	<.001
**PHQ8 response time (minutes)**	107.42 [40.78, 320.76]	123.17 [35.15, 246.15]	107.89 [37.38, 329.76]	174.82 [63.87, 261.17]	.81
**PHQ8 completion time (seconds)**	51.78 [41.09, 66.86]	47.00 [37.99, 61.73]	46.49 [37.06, 61.44]	55.58 [41.17, 75.53]	.16
**RSES response time (minutes)**	105.32 [33.88, 336.49]	104.53 [21.69, 290.55]	83.92 [33.37, 280.32]	178.30 [64.61, 336.32]	.55
**RSES completion time (seconds)**	57.42 [45.97, 74.53]	53.36 [43.56, 68.84]	50.79 [41.02, 72.28]	64.11 [48.09, 97.14]	.06
**Site, n (%)**					.007
CIBER	26 (21.3)	15 (28.3)	17 (27.9)	33 (42.9)	
KCL	92 (75.4)	35 (66.0)	37 (60.7)	42 (54.5)	
VUMC	4 (3.3)	3 (5.7)	7 (11.5)	2 (2.6)	
**Married Status, n (%)**					.85
Single/separated/divorced/widowed	60 (49.2)	24 (45.3)	30 (49.2)	41 (53.2)	
Married/cohabiting/LTR	62 (50.8)	29 (54.7)	31 (50.8)	36 (46.8)	
**Years in education**	14.50 [12.00, 18.00]	17.00 [13.00, 18.00]	16.00 [13.00, 19.00]	15.00 [12.00, 19.00]	.55
**Having children, n (%)**	69 (56.6)	26 (49.1)	35 (57.4)	43 (55.8)	.85
**Employed, n (%)**	52 (42.6)	24 (45.3)	29 (47.5)	30 (39.0)	.89
**Annual income, n (%)**					.15
<15,000 (£/€)	32 (26.2)	14 (26.4)	15 (24.6)	25 (32.5)	
15,000-55,000 (£/€)	69 (56.6)	29 (54.7)	39 (63.9)	44 (57.1)	
more than 55000 (£/€)	21 (17.2)	8 (15.1)	7 (11.5)	8 (10.4)	
**Accommodation, n (%)**					.82
Own outright/with mortgage	71 (58.2)	32 (60.4)	33 (54.1)	41 (53.2)	
Renting	41 (33.6)	18 (34.0)	23 (37.7)	28 (36.4)	
Living rent-free	10 (8.2)	3 (5.7)	4 (6.6)	6 (7.8)	
**Baseline PHQ8 score**	8.50 [5.25, 14.00]	11.00 [6.00, 17.00]	10.50 [7.25, 15.75]	13.00 [9.00, 17.00]	.02
**Having comorbidities, n (%)**	74 (60.7)	26 (49.1)	33 (54.1)	43 (55.8)	.53
**Taking depression medication, n (%)**	73 (59.8)	36 (67.9)	44 (72.1)	55 (71.4)	.24
**Number of contact logs**	10.50 [7.00, 17.00]	9.00 [6.00, 14.00]	10.00 [6.00, 15.00]	6.00 [3.00, 10.00]	<.001
**Provided phone, n (%)**	39 (32.0)	16 (30.2)	16 (26.2)	9 (11.8)	.01
**Brand of smartphone, n (%)**					<.001
Motorola	81 (66.9)	22 (41.5)	19 (31.1)	14 (18.4)	
Samsung	17 (14.0)	11 (20.8)	19 (31.1)	43 (56.6)	
Other	23 (19.0)	20 (37.7)	23 (37.7)	19 (25.0)	

Supplementary Table 10. A summary of participants' s characteristics across four distinct engagement subgroups of Fitbit-Passive data for the first 94 weeks of the RADAR-MDD study. The median and interquartile range (IQR) of continuous variables and the count number and percentage of category variables are reported in the table. All p values were assessed using the Kruskal-Wallis tests.

Characteristics	C1	C2	C3	C4	p
Number of participants, n	153	58	52	50	
Age	53.00 [40.00, 60.00]	45.00 [32.00, 54.75]	43.00 [30.50, 53.00]	54.50 [40.75, 63.00]	<.001
Male, n (%)	38 (24.8)	18 (31.0)	10 (19.2)	12 (24.0)	.56
Days with Fitbit passive data	634.00 [586.00, 655.00]	426.50 [358.25, 480.00]	218.00 [162.75, 264.00]	61.50 [2.50, 100.50]	<.001
PHQ8 response time (minutes)	106.57 [38.25, 252.87]	166.12 [59.78, 305.03]	198.13 [39.73, 378.89]	160.82 [63.15, 259.01]	.56
PHQ8 completion time (seconds)	47.46 [39.41, 58.66]	48.08 [39.29, 63.49]	57.93 [41.96, 77.13]	67.27 [48.78, 92.50]	.001
RSES response time (minutes)	100.70 [27.77, 234.81]	143.48 [38.42, 316.74]	180.24 [18.92, 425.51]	149.08 [50.24, 331.34]	.56
RSES completion time (seconds)	52.44 [43.61, 66.07]	53.18 [42.18, 73.52]	67.99 [48.09, 94.11]	71.15 [54.14, 106.58]	.001
Site, n (%)					.11
CIBER	35 (22.9)	15 (25.9)	20 (38.5)	21 (42.0)	
KCL	109 (71.2)	39 (67.2)	30 (57.7)	28 (56.0)	
VUMC	9 (5.9)	4 (6.9)	2 (3.8)	1 (2.0)	
Married Status, n (%)					.39
Single/separated/divorced/widowed	70 (45.8)	34 (58.6)	27 (51.9)	24 (48.0)	
Married/cohabiting/LTR	83(54.2)	24 (41.4)	25 (48.1)	26 (52.0)	
Years in education	16.00 [12.00, 19.00]	15.00 [13.00, 18.00]	15.00 [12.00, 20.00]	14.00 [11.00, 17.00]	.19
Having children, n (%)	84 (54.9)	27 (46.6)	26 (50.0)	36 (72.0)	.17
Employed, n (%)	74 (48.4)	24 (41.4)	18 (34.6)	19 (38.0)	.27
Annual income, n (%)					.32
<15,000 (£/€)	37 (24.2)	17 (29.3)	17 (32.7)	15 (30.0)	
15,000-55,000 (£/€)	87 (56.9)	34 (58.6)	31 (59.6)	29 (58.0)	
more than 55000 (£/€)	29 (19.0)	6 (10.3)	4 (7.7)	5 (10.0)	
Accommodation, n (%)					.08
Own outright/with mortgage	94 (61.4)	26 (44.8)	23 (44.2)	34 (68.0)	
Renting	50 (32.7)	23 (39.7)	24 (46.2)	13 (26.0)	
Living rent-free	8 (5.2)	8 (13.8)	5 (9.6)	2 (4.0)	
Baseline PHQ8 score	9.00 [7.00, 13.75]	12.00 [6.00, 18.00]	13.00 [7.00, 17.00]	13.00 [8.75, 17.00]	.11
Having comorbidities, n (%)	81 (52.9)	31 (53.4)	27 (51.9)	37 (74.0)	.05
Taking depression medication, n (%)	102 (66.7)	37 (63.8)	37 (71.2)	32 (64.0)	.84
Number of contact logs	10.00 [7.00, 16.00]	8.00 [5.00, 14.00]	9.00 [5.75, 12.25]	6.00 [3.00, 9.75]	<.001
Provided phone, n (%)	40 (26.1)	16 (27.6)	12 (23.1)	12 (24.5)	.95
Brand of smartphone, n (%)					.45
Motorola	72 (47.4)	27 (46.6)	20 (38.5)	17 (34.7)	
Samsung	39 (25.7)	17 (29.3)	17 (32.7)	12 (24.5)	
Other	41 (27.0)	14 (24.1)	15 (28.8)	20 (40.8)	

Supplementary Table 11. A summary of ethnicity difference across three distinct engagement subgroups of Phone-Active, Phone-Passive, and Fitbit-Passive data streams for the first 43 weeks of the RADAR-MDD study (ethnicity data was available for KCL and VUMC sites). All p values were assessed using the Kruskal-Wallis tests.

**Data stream**	**C1**	**C2**	**C3**	**p value**
**Phone-Active**				<.001
White	175 (95.1%)	110 (84.0%)	119 (77.8%)	
Black	3 (1.6%)	3 (2.3%)	8 (5.2%)	
Asian	2 (1.1%)	2 (1.5%)	12 (7.8%)	
Other	4 (2.2%)	16 (12.2%)	14 (9.2%)	
**Phone-Passive**				.001
White	174 (87.4%)	115 (92.7%)	115 (79.3%)	
Black	10 (5.0%)	1 (0.8%)	3 (2.1%)	
Asian	2 (1.0%)	4 (3.2%)	10 (6.9%)	
Other	13 (6.5%)	4 (3.2%)	17 (11.7%)	
**Fitbit-Passive**				.003
White	296 (89.7%)	57 (81.4%)	51 (75.0%)	
Black	9 (2.7%)	4 (5.7%)	1 (1.5%)	
Asian	6 (1.8%)	4 (5.7%)	6 (8.8%)	
Other	19 (5.8%)	5 (7.1%)	10 (14.7%)	

Supplementary Table 12. A list of 19 comorbidities that recorded at the enrollment of the RADAR-MDD study.

**Number**	**Comorbidity**
1	Asthma
2	Chronic bronchitis
3	Other chest trouble
4	Diabetes
5	Stomach or other digestive disorder
6	Liver trouble
7	Kidney trouble
8	Rheumatoid arthritis
9	Osteoarthritis
10	Heart trouble
11	Cancer
12	High blood pressure
13	Multiple Sclerosis
14	Epilepsy/fits
15	Stroke
16	Other neurological trouble
17	Migraine
18	Back trouble
19	Other

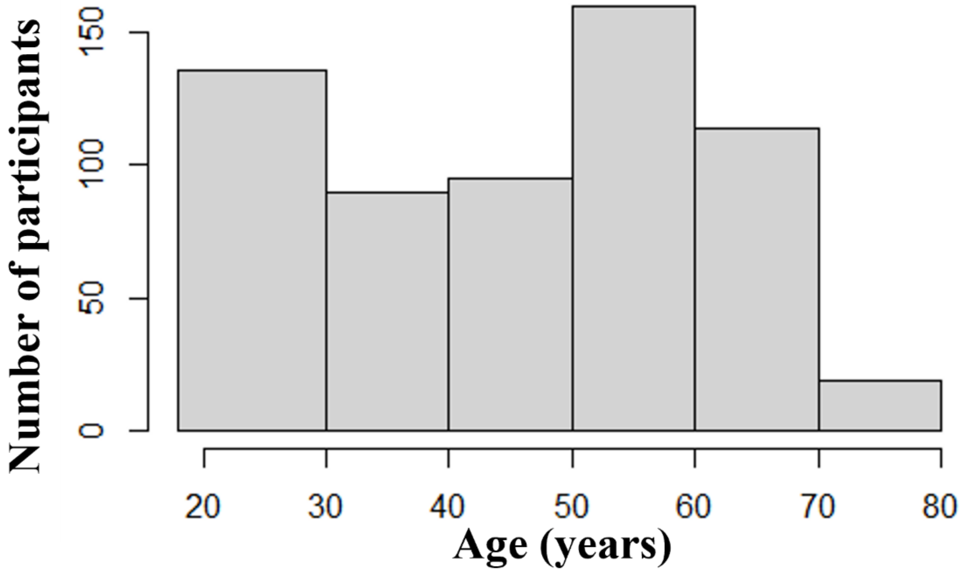

Supplementary Figure 1. The histogram of age distribution for 614 participants in the RADAR-MDD study.

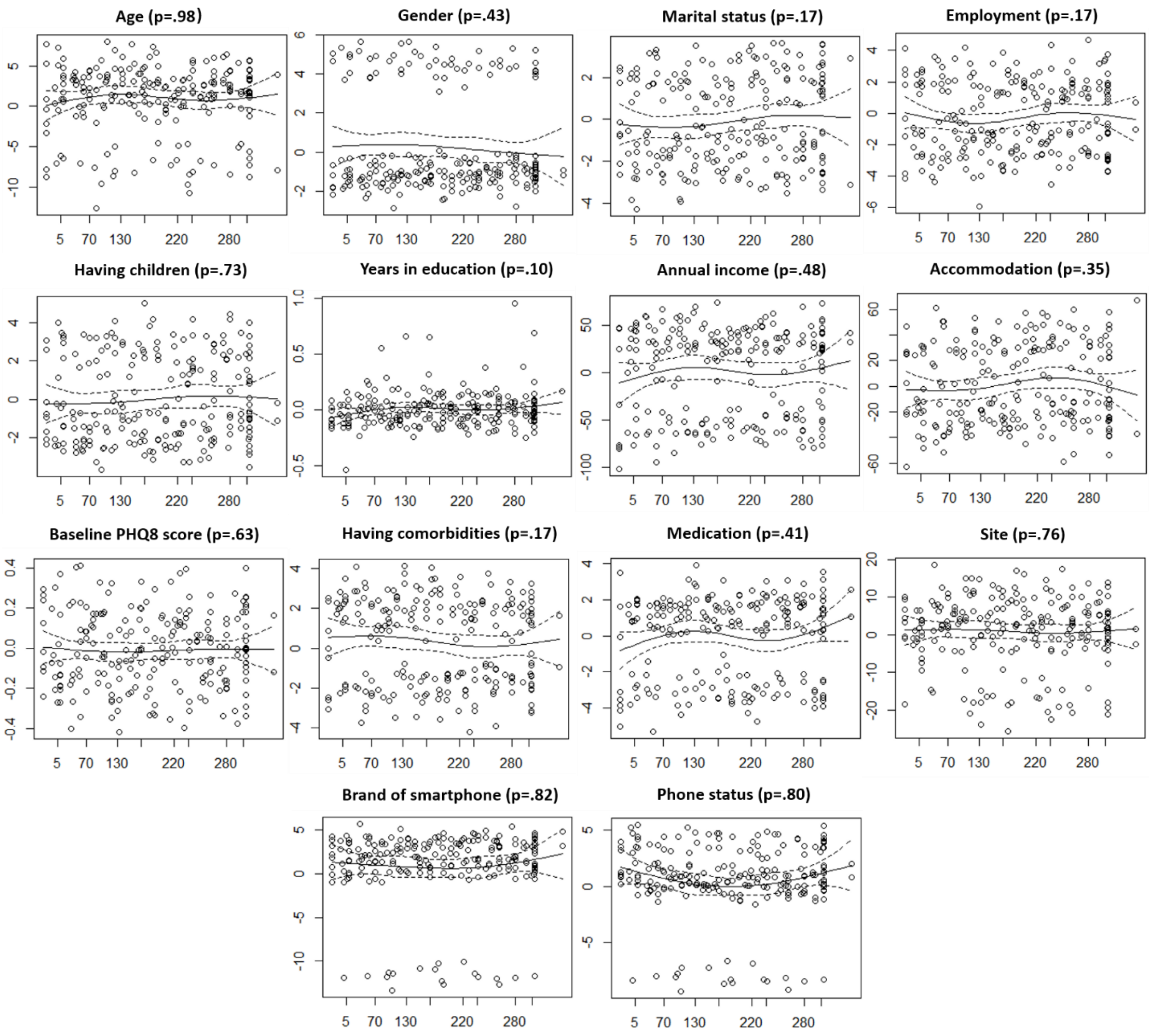

Supplementary Figure 2. Scaled Schoenfeld residual plots for all variables in Cox Proportional-Hazard model for the Phone-Active data stream for the first 43 weeks of the study. The results of proportional hazard assumption tests are shown in Supplement Table 2. All variables passed the assumption test.

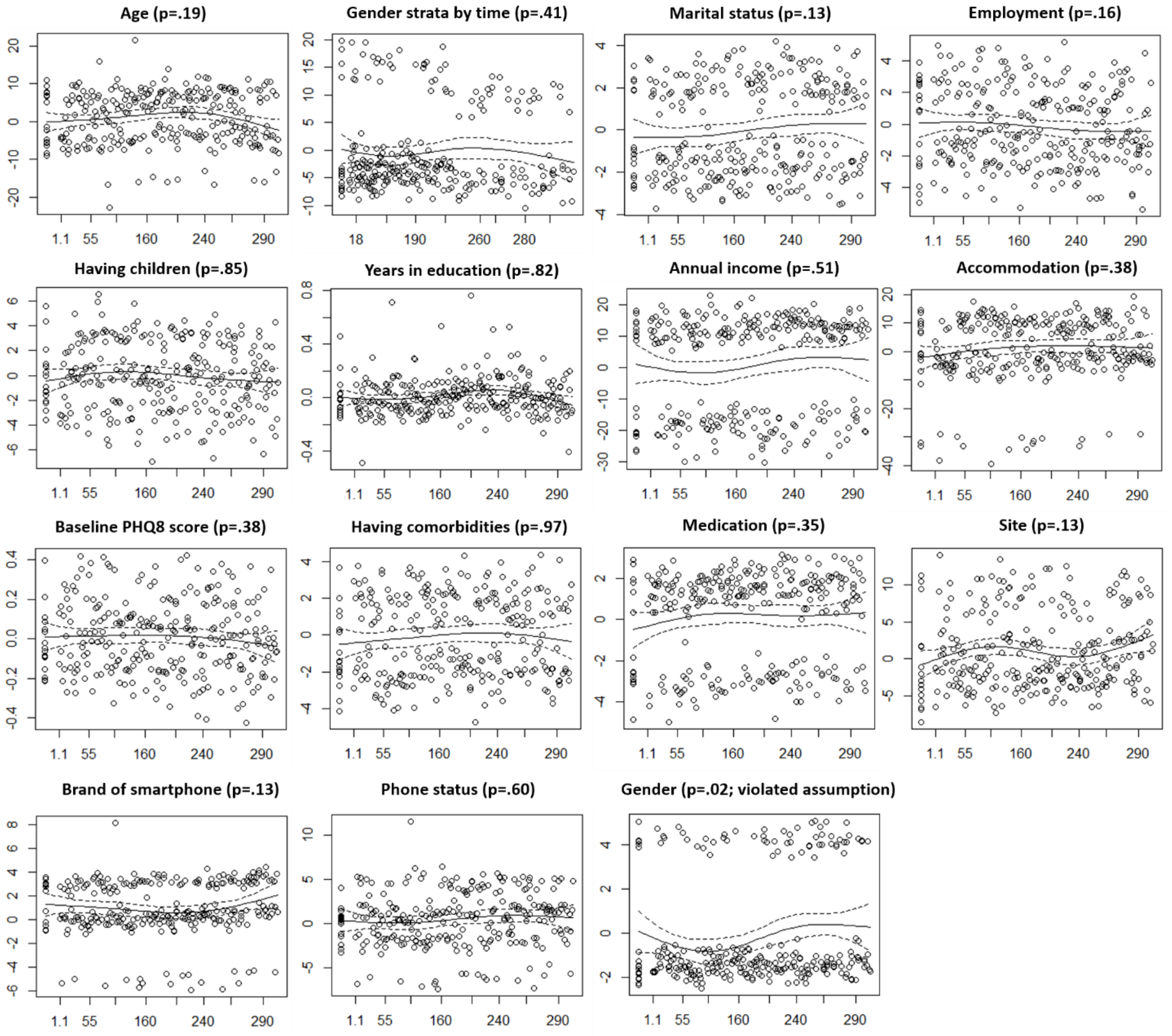

Supplementary Figure 3. Scaled Schoenfeld residual plots for all variables in Cox Proportional-Hazard model for the Phone-Passive data stream for the first 43 weeks of the study. The results of proportional hazard assumption tests are shown in Supplement Table 2. An interaction term of the "gender" variable with a split time variable (cut points are 130 and 240) was used to make the variable meet the model assumption. The previous scaled Schoenfeld residual plot (violated the assumption) of "gender" is shown in the last subplot.

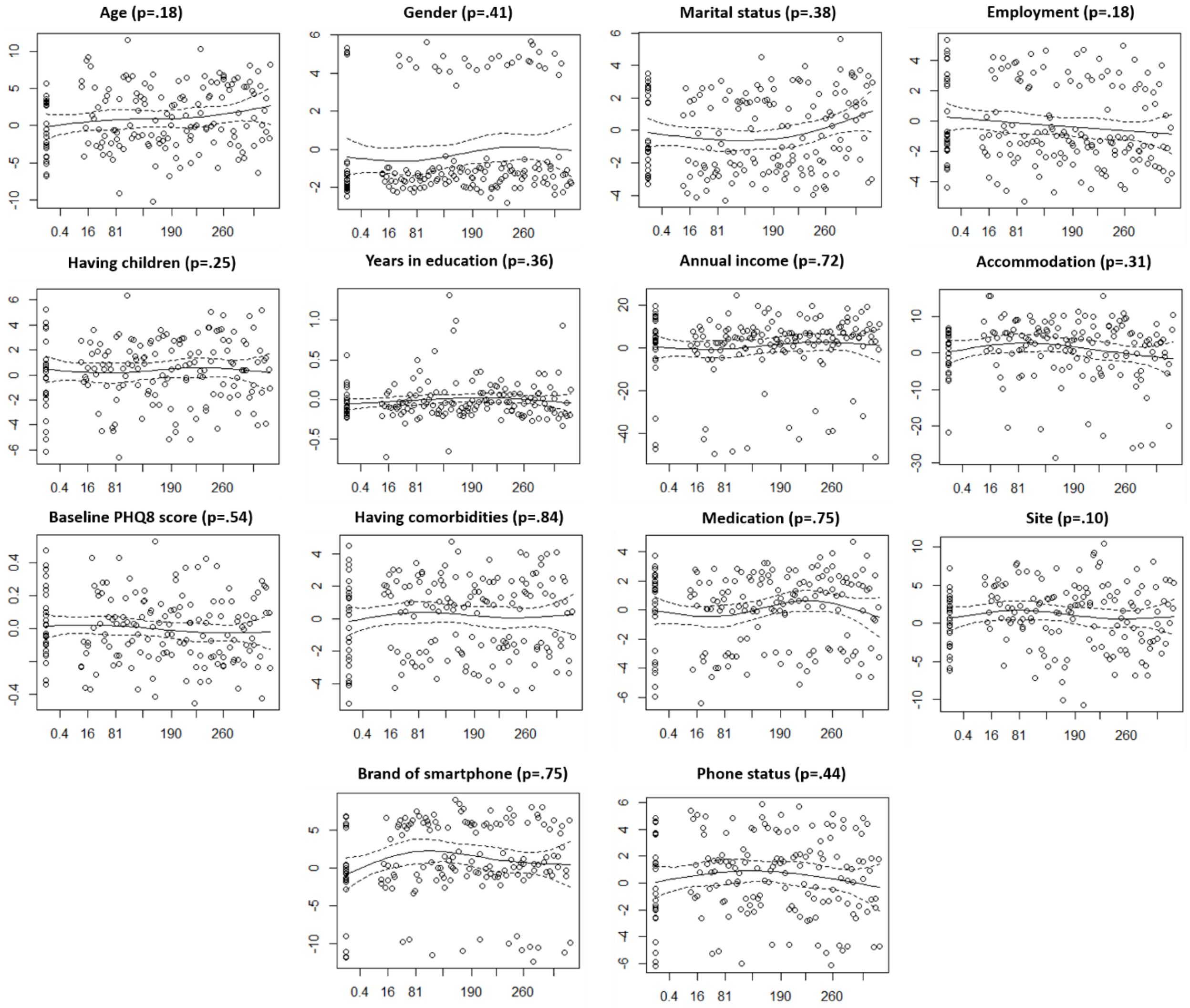

Supplementary Figure 4. Scaled Schoenfeld residual plots for all variables in Cox Proportional-Hazard model for the Fitbit-Passive data stream for the first 43 weeks of the study. The results of proportional hazard assumption tests are shown in Supplement Table 2. All variables passed the assumption test.

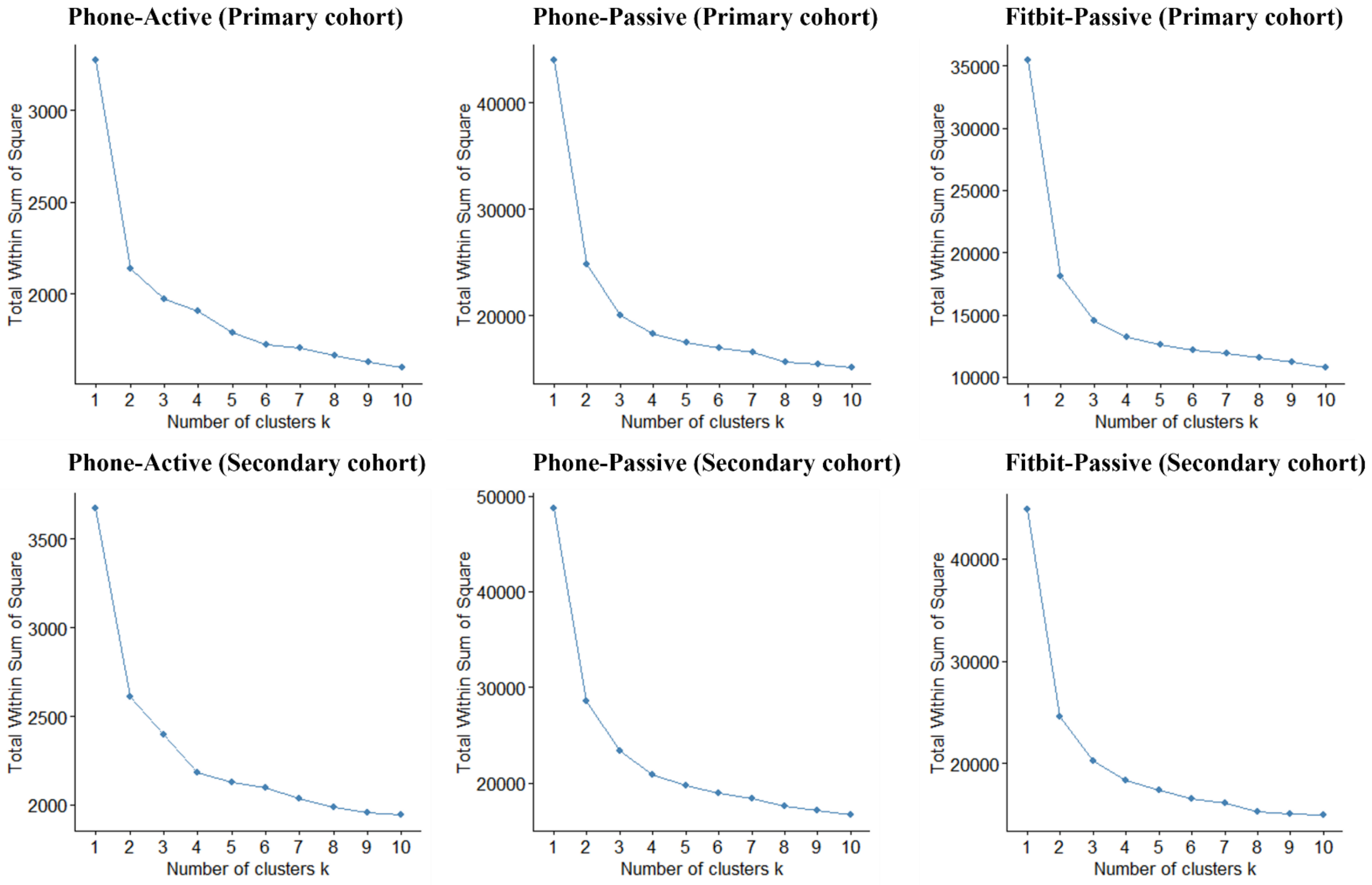

Supplementary Figure 5. Comparison of within-cluster variations across data streams using different cluster sizes (N=1-10) for K-means clustering. The optimal numbers of clusters for primary (43-week observation period) and secondary (94-week observation period) cohorts are 3 and 4, respectively.

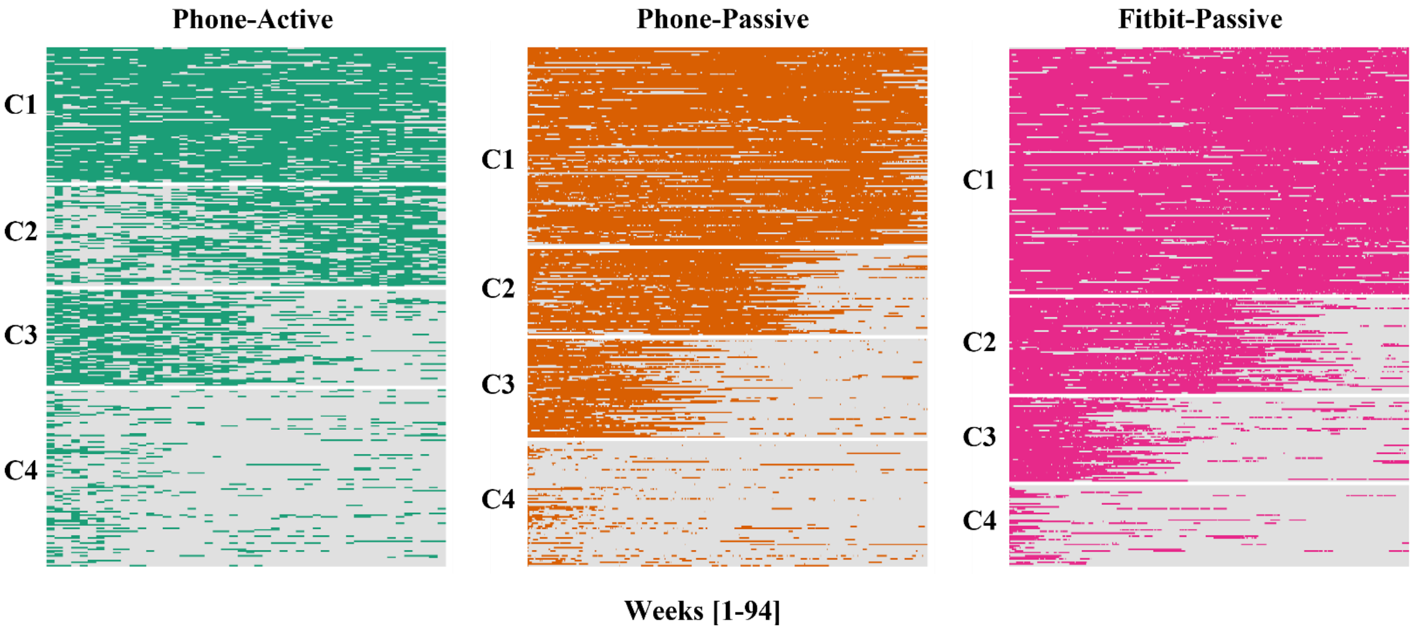

Supplementary Figure 6. Heatmaps of participant longitudinal engagement patterns for the three data streams in the longer observation period (94 weeks), clustered using K-means clustering. In each heatmap, each row represents a data-availability vector of one participant (described in Methods), and subgroups were arranged from the most engaged cluster to the least engaged cluster (C1-C4).
