# Supplementary material for: Long-term participant retention and engagement patterns in an app and wearable-based multinational remote digital depression study
Source: NPJ Digit Med. 2023 Feb 17;6:25. doi: 10.1038/s41746-023-00749-3 (PMC9938183; doi:10.1038/s41746-023-00749-3)
Supplement: Supplementary file 2 — Reporting Summary [file 41746_2023_749_MOESM2_ESM.pdf]

nature portfolio

Corresponding author(s): Richard Dobson and Abhishek Pratap

Last updated by author(s): Sep 8, 2022

Reporting Summary

Nature Portfolio wishes to improve the reproducibility of the work that we publish. This form provides structure for consistency and transparency in reporting. For further information on Nature Portfolio policies, see our Editorial Policies and the Editorial Policy Checklist.

Statistics

For all statistical analyses, confirm that the following items are present in the figure legend, table legend, main text, or Methods section.

n/a	Confirmed	
☐	☒	The exact sample size (n) for each experimental group/condition, given as a discrete number and unit of measurement
☐	☒	A statement on whether measurements were taken from distinct samples or whether the same sample was measured repeatedly
☐	☒	The statistical test(s) used AND whether they are one- or two-sided<br>*Only common tests should be described solely by name; describe more complex techniques in the Methods section.*
☐	☒	A description of all covariates tested
☐	☒	A description of any assumptions or corrections, such as tests of normality and adjustment for multiple comparisons
☐	☒	A full description of the statistical parameters including central tendency (e.g. means) or other basic estimates (e.g. regression coefficient) AND variation (e.g. standard deviation) or associated estimates of uncertainty (e.g. confidence intervals)
☐	☒	For null hypothesis testing, the test statistic (e.g. F, t, r) with confidence intervals, effect sizes, degrees of freedom and P value noted<br>*Give P values as exact values whenever suitable.*
☒	☐	For Bayesian analysis, information on the choice of priors and Markov chain Monte Carlo settings
☒	☐	For hierarchical and complex designs, identification of the appropriate level for tests and full reporting of outcomes
☐	☒	Estimates of effect sizes (e.g. Cohen's d, Pearson's r), indicating how they were calculated

Our web collection on statistics for biologists contains articles on many of the points above.

Software and code

Policy information about availability of computer code

Data collection	The data in this study was collected using RADAR-base platform. The code for the platform is available through a GitHub code repository (https://github.com/RADAR-base).
Data analysis	The complete code used for the analysis is available through a GitHub code repository (https://github.com/kcl-bhi/RADAR_MDD_Pariticipant_Engagement_and_Retention_Analysis).

For manuscripts utilizing custom algorithms or software that are central to the research but not yet described in published literature, software must be made available to editors and reviewers. We strongly encourage code deposition in a community repository (e.g. GitHub). See the Nature Portfolio guidelines for submitting code & software for further information.

Data

Policy information about availability of data

All manuscripts must include a data availability statement. This statement should provide the following information, where applicable:

- Accession codes, unique identifiers, or web links for publicly available datasets
- A description of any restrictions on data availability
- For clinical datasets or third party data, please ensure that the statement adheres to our policy

The datasets used for the present study can be made available through reasonable requests to the RADAR-CNS consortium. Please email the corresponding author for details.

Human research participants

Policy information about studies involving human research participants and Sex and Gender in Research.

Reporting on sex and gender	Gender information was self-reported via surveys in our study. Gender was considered as a covariate of our participant retention and engagement analysis.
Population characteristics	The cohort's median (IQR) age was 49 (32.0, 58.8) years (ranges from 18 to 80 years old) with a median baseline PHQ8 score of 10 (7.0, 16.0). All participants in this study had a history of recurrent MDD with at least one episode within the last 2 years that meets DSM-5 diagnostic criteria for diagnosis of MDD.
Recruitment	Potentially eligible individuals will be identified either through existing research cohorts (in London and The Netherlands) who have given consent to be contacted for research purposes, or through mental health services (in London and Barcelona). These individuals will be contacted by a member of the on-site research teams and sent the study information sheet and consent from. Those who provide verbal consent to participate will have a more detailed conversation with the research teams, during which they will have an opportunity to ask questions about the study, have their eligibility con- firmed, and arrange a suitable time for enrolment.
Ethics oversight	Ethical approval has been obtained in London from the Camberwell St Giles Research Ethics Committee (REC reference: 17/LO/1154), in Spain from the CEIC Fundacio Sant Joan de Deu (CI: PIC-128-17) and in the Netherlands from the Medische Ethische Toetsingscommissie VUmc (METc VUmc registratienummer: 2018.012 – NL63557.029.17).

Note that full information on the approval of the study protocol must also be provided in the manuscript.

Field-specific reporting

Please select the one below that is the best fit for your research. If you are not sure, read the appropriate sections before making your selection.

☐ Life sciences ☒ Behavioural & social sciences ☐ Ecological, evolutionary & environmental sciences

For a reference copy of the document with all sections, see nature.com/documents/nr-reporting-summary-flat.pdf

Behavioural & social sciences study design

All studies must disclose on these points even when the disclosure is negative.

Study description	The data used in the present study are quantitative survey data and quantitative passive data from phones and wearables.
Research sample	Participants in the present study are adults with depression history recruited from the UK, Spain, and the Netherlands. The cohort's median (IQR) age was 49 (32.0, 58.8) years with the majority being females (75.7%). Since the aim of this study was to investigate whether mobile technologies can predict depression relapse, one of the inclusion criteria is participants in this study had a history of recurrent MDD with at least one episode within the last 2 years that meets DSM-5 diagnostic criteria for diagnosis of MDD.
Sampling strategy	The study aimed to collect 100 depression relapse cases. Since an approximated annual relapse of depression rate is 33%, 300 participants would be required, followed-up for one year. Also the research team considered participant attrition in remote study, therefore, the study aimed to recruit 600 participants.
Data collection	Participants were asked to regularly complete self-reported surveys via the active app. Additionally, participants' real-world behavior was gathered passively using the Android passive monitoring app and a Fitbit wearable.
Timing	Data was collected between November 2017 to April 2021.
Data exclusions	Nine participants recruited from a second site in Spain were not included in the present analysis due to the small sample size.
Non-participation	114 of 623 participants declined participation for different reasons.
Randomization	Participants were not allocated into experimental groups.

Reporting for specific materials, systems and methods

We require information from authors about some types of materials, experimental systems and methods used in many studies. Here, indicate whether each material, system or method listed is relevant to your study. If you are not sure if a list item applies to your research, read the appropriate section before selecting a response.

Materials & experimental systems

n/a	Involved in the study
☒	☐ Antibodies
☒	☐ Eukaryotic cell lines
☒	☐ Palaeontology and archaeology
☒	☐ Animals and other organisms
☒	☐ Clinical data
☒	☐ Dual use research of concern

Methods

n/a	Involved in the study
☒	☐ ChIP-seq
☒	☐ Flow cytometry
☒	☐ MRI-based neuroimaging
